# A photosynthesis operon in the chloroplast genome drives speciation in evening primroses

**DOI:** 10.1093/plcell/koab155

**Published:** 2021-05-28

**Authors:** Arkadiusz Zupok, Danijela Kozul, Mark Aurel Schöttler, Julia Niehörster, Frauke Garbsch, Karsten Liere, Axel Fischer, Reimo Zoschke, Irina Malinova, Ralph Bock, Stephan Greiner

**Affiliations:** Department Organelle Biology, Biotechnology and Molecular Ecophysiology, Max Planck Institute of Molecular Plant Physiology, Potsdam-Golm, D-14476, Germany; Department Organelle Biology, Biotechnology and Molecular Ecophysiology, Max Planck Institute of Molecular Plant Physiology, Potsdam-Golm, D-14476, Germany; Department Organelle Biology, Biotechnology and Molecular Ecophysiology, Max Planck Institute of Molecular Plant Physiology, Potsdam-Golm, D-14476, Germany; Department Organelle Biology, Biotechnology and Molecular Ecophysiology, Max Planck Institute of Molecular Plant Physiology, Potsdam-Golm, D-14476, Germany; Department Organelle Biology, Biotechnology and Molecular Ecophysiology, Max Planck Institute of Molecular Plant Physiology, Potsdam-Golm, D-14476, Germany; Institut für Biologie/Molekulare Genetik, Humboldt-Universität zu Berlin, Berlin, D-10115, Germany; Department Organelle Biology, Biotechnology and Molecular Ecophysiology, Max Planck Institute of Molecular Plant Physiology, Potsdam-Golm, D-14476, Germany; Department Organelle Biology, Biotechnology and Molecular Ecophysiology, Max Planck Institute of Molecular Plant Physiology, Potsdam-Golm, D-14476, Germany; Department Organelle Biology, Biotechnology and Molecular Ecophysiology, Max Planck Institute of Molecular Plant Physiology, Potsdam-Golm, D-14476, Germany; Department Organelle Biology, Biotechnology and Molecular Ecophysiology, Max Planck Institute of Molecular Plant Physiology, Potsdam-Golm, D-14476, Germany; Department Organelle Biology, Biotechnology and Molecular Ecophysiology, Max Planck Institute of Molecular Plant Physiology, Potsdam-Golm, D-14476, Germany

## Abstract

Genetic incompatibility between the cytoplasm and the nucleus is thought to be a major factor in species formation, but mechanistic understanding of this process is poor. In evening primroses (*Oenothera* spp.), a model plant for organelle genetics and population biology, hybrid offspring regularly display chloroplast–nuclear incompatibility. This usually manifests in bleached plants, more rarely in hybrid sterility or embryonic lethality. Hence, most of these incompatibilities affect photosynthetic capability, a trait that is under selection in changing environments. Here we show that light-dependent misregulation of the plastid *psbB* operon, which encodes core subunits of photosystem II and the cytochrome *b*_6_*f* complex, can lead to hybrid incompatibility, and this ultimately drives speciation. This misregulation causes an impaired light acclimation response in incompatible plants. Moreover, as a result of their different chloroplast genotypes, the parental lines differ in photosynthesis performance upon exposure to different light conditions. Significantly, the incompatible chloroplast genome is naturally found in xeric habitats with high light intensities, whereas the compatible one is limited to mesic habitats. Consequently, our data raise the possibility that the hybridization barrier evolved as a result of adaptation to specific climatic conditions.

## Teaser

Photosynthesis genes encoded on the chloroplast genome establish hybridization barriers.

## Introduction

Incompatibility between the nuclear and organellar genomes represents a mechanism of reproductive isolation that is observed in a wide range of taxa (Levin, 2003; Chou and Leu, 2010; Greiner et al., 2011; Burton et al., 2013; Greiner and Bock, 2013; Sloan et al., 2017; Fishman and Sweigart, 2018; Bogdanova, 2020; Postel and Touzet, 2020). However, with the exception of cytoplasmic male sterility, a commercially important trait, little is known about the molecular and evolutionary mechanisms of cytoplasmic incompatibility (CI). Mechanistic studies are available from only a few cases (Schmitz-Linneweber et al., 2005; Harrison and Burton, 2006; Lee et al., 2008; Burton et al., 2013; Meiklejohn et al., 2013; Fishman and Sweigart, 2018), in contrast with the great biological importance of the phenomenon. Increasing evidence is accumulating that CI arises early in the separation of two genetic lineages (Levin, 2003; Fishman and Willis, 2006; Greiner et al., 2011; Burton et al., 2013; Barnard-Kubow et al., 2016; Postel and Touzet, 2020), and thus represents an initial barrier leading to reproductive isolation. This suggests that CI is a major determinant of species formation, highlighting the need for a mechanistic understanding of its molecular basis in the context of population genetics.

**Figure:**
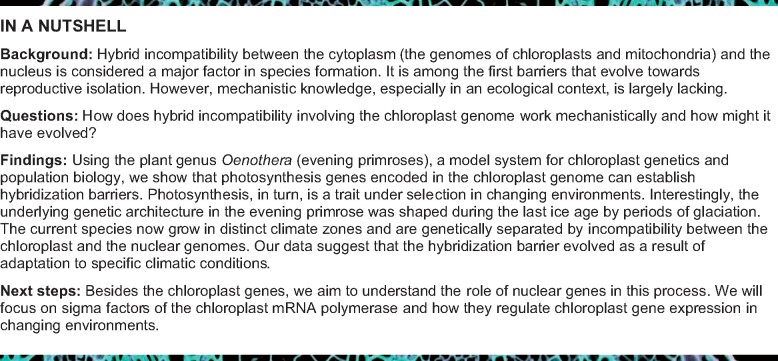


**Figure 1:**
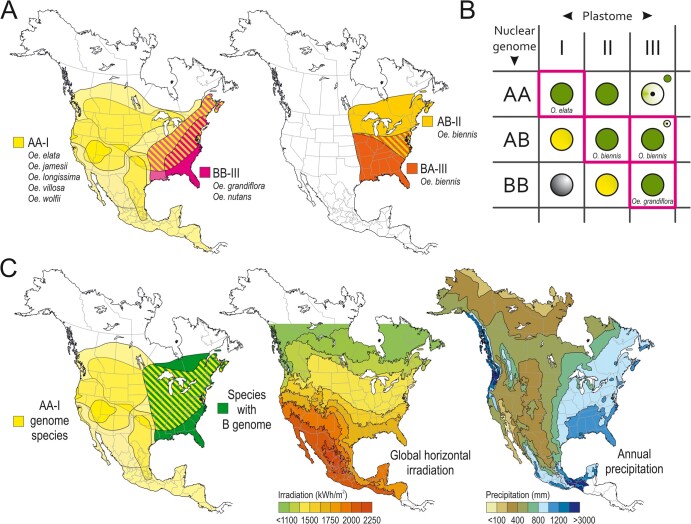
Distribution of *Oenothera* AA-I, BB-III and AB-II/BA-III species, and compatibility/incompatibility relations upon hybridization. A, Native distribution of *Oenothera* A and B genome species (pure colors) and their hybridization zones (two-color pattern). Yellow and magenta gradients indicate occurrence of distinct species or subspecies. B, Genetic species concept of evening primroses, based on plastome/nuclear genome compatibility and incompatibility, exemplified for the A and B genome species. Species are defined by their combinations of nuclear and chloroplast genomes (boxed in magenta), and are genetically separated by PGIs that occur upon hybridization and vary in the severity of the hybrid phenotype (BB-I white, AB-I and BB-II yellow-green, and AA-III bleaching leaf phenotype). C, Association of AA-I and B genome (BB and AB) species of *Oenothera* to the xeric and mesic habitats of North and Central America. Distribution maps redrawn from Dietrich et al. (1997). Climate data are from SolarGis and North American Environmental Atlas.

The evening primrose (*Oenothera*) is a model plant that is uniquely suited to address the mechanisms of reproductive isolation via hybrid incompatibility. Crosses between *Oenothera* species usually produce viable offspring that regularly display incompatibility between the chloroplast and the nuclear genomes (plastome–genome incompatibility [PGI]). These incompatibilities usually manifest in bleached plants, more rarely in hybrid sterility or embryonic lethality. They represent the only strong hybridization barrier between *Oenothera* species, which often co-occur in overlapping ecological niches within hybridization zones (Stubbe, 1964; Dietrich et al., 1997; Greiner et al., 2011; Figure 1). Hybrid incompatibility of nuclear loci is essentially absent (Stubbe and Raven, 1979b; Greiner and Köhl, 2014), underscoring the importance of CI as the cause of incipient isolation of the hybrid. In addition, *Oenothera* is a prime example for hybrid speciation (Cleland, 1957; Hollister et al., 2019), in that permanent translocation heterozygosity, a form of cross-inducible functional asexuality, can occur. Such genotypes display a meiotic ring and breed true upon self-fertilization. In crosses, this can lead to an immediate fixation of a hybrid (Cleland, 1972; Harte, 1994; Rauwolf et al., 2008; Golczyk et al., 2014) (see “Methods” and below).

In evening primroses (section *Oenothera* subsection *Oenothera*), based on phenotype, phylogeography, and behavior in crossings, three genetic lineages (A, B, and C) exist that are genetically separated by PGI (Stubbe, 1964; Cleland, 1972; Greiner et al., 2011). These genetic lineages occur in either homozygous (AA, BB, or CC) or in a stable heterozygous (AB, AC, or BC) state. They can be combined with ﬁve basic chloroplast genome types (I–V), which differ in their compatibility relations to the nuclear genotypes (Figure 1B).

The presence of distinct nuclear and chloroplast genomes and the sexual separation of the species by PGI have led to the development of a genetic species concept describing 13 species for *Oenothera* (Stubbe, 1964; Raven et al., 1979; Stubbe and Raven, 1979b; Dietrich et al., 1997). This species concept (of the so-called American school) is in sharp contrast to the largely morphological species definition of the European school that recognizes about 80 species (Rostański, 1982; 1985; Rostański et al., 2010; Woźniak-Chodacka, 2018). According to the American school, however, specific combinations of nuclear and chloroplast genomes define the species (Dietrich et al., 1997). Other genome combinations can occur in weak or inviable hybrids, thus sexually separating the species (Figure 1B). Strikingly, these chloroplast-mediated speciation barriers rely on photosynthesis, a trait that is under selection in changing environmental conditions (Arntz and Delph, 2001; Flood, 2019). This makes *Oenothera* an appealing model to understand the genetic basis of speciation. The incompatibility loci causing separation of the species are part of the species concept and, hence, relevant for speciation by definition. They may even be a result of adaptive evolution.

For example, hybridization between *Oenothera elata* (an AA-I species) and *Oenothera grandiflora* (a BB-III species) produces the incompatible combination AB-I that displays a bleached-leaf phenotype. This genetic incompatibility creates the major hybridization barrier between AA and BB species that appears to prevent colonization of western North America by B genome species (Figure 1C; Greiner et al., 2011). It should be emphasized that hybridization is frequent in the genus in nature. As a rule, hybridization occurs if plastome–genome combinations permit it. Importantly, all viable combinations can be confirmed in hybrids in nature (Dietrich et al., 1997). Hence, PGI appears to act as a major mechanism preventing gene flow. In addition, ecological species separation is likely to occur. For example, existence of the green and compatible hybrid AA-II can be confirmed, but it does not establish stable populations in nature (Dietrich et al., 1997; Figure 1, A and B).

It is assumed that climate changes and periods of glaciation during the Pleistocene have shaped the genetic and ecological characteristics of the basic lineages A, B, and C in *Oenothera* (Cleland, 1972). This is well supported both by the estimated divergence time of the chloroplast genomes (Greiner et al., 2008b) and by nuclear genome variation (Levy and Levin, 1975; Hollister et al., 2015). Following this view, the three lineages originated from Middle America and reached the North American continent in several waves. The current lineages resemble the ancestral sexual and homozygous species AA-I, BB-III, and CC-V, and crosses between them usually result in PGI (Stubbe, 1964; Greiner et al., 2011). However, during the Pleistocene, hybridization between the basic lineages must have happened that produced viable offspring (Cleland, 1957, 1972; Dietrich et al., 1997). Those were fixed in the structural heterozygous and functional asexual species (AB-II or BA-III, AC-IV, and BC-IV). Hence, plastomes II and IV can be seen as relict genotypes of earlier stages of plastome evolution (Stubbe, 1964; Dietrich et al., 1997). Interestingly, those relict genotypes are now quite likely maintained in adaptive niches, since they are not outcompeted by the more recent, faster multiplying and more aggressive, chloroplast genomes (Stubbe, 1963, 1964):

Biparental chloroplast inheritance, which is the rule in evening primrose, leads to selfish evolution of aggressive or more competitive (e.g. faster multiplying) chloroplast genotypes upon hybridization (Grun, 1976; Greiner et al., 2015; Sobanski et al., 2019). In *Oenothera*, the most competitive chloroplasts are fixed as current evolutionary end points in the homozygote lineages AA-I, BB-III, and CC-V. These competitive chloroplast genotypes (I, III, and V) have the potential to outcompete the weaker ones (II and IV) in hybrid genotypes. For example, the common evening primrose (*Oenothera biennis*) harbors plastomes II and III in overlapping subpopulations (Cleland, 1972; Dietrich et al., 1997). Since plastids with plastome III are more aggressive than those with plastome II, they should outcompete plastome II in this species. Observed, however, is that separation of plastomes II and III in the two major subpopulations coincides with the expansion of the Wisconsin glacier (see Supplemental Figure S3 of Anstett et al., 2015; Figure 1A). This suggests that post-glaciation dispersal events and the presence of an ecological niche maintain the less aggressive chloroplast type in the northern parts of the *O. biennis* population.

Taken together, even recent plastome divergence in evening primrose appears to be a consequence of glaciation that is fixed by an interplay of adaptive and selfish evolution. The influence of genetic drift on hybrid incompatibly among the *Oenothera* populations is currently unknown.

Our aim was to understand the mechanism of the AB-I incompatibility that genetically separates the A and B lineages (Figure 1). We were further asking to what extent the underlying incompatibility might have evolved as a result of adaptive divergence in the two lineages. Based on association mapping in the chloroplast genome, the dual promoter region in the intergenic spacer between the *clpP* operon and the *psbB* operon was proposed to be involved in the incompatibility AB-I (Greiner et al., 2008a). The *clpP* gene encodes the proteolytic subunit of the Clp protease, the *psbB* operon encodes core subunits of photosystem II (PSII) and the cytochrome *b*_6_*f* complex (Cyt*b*_6_*f*). However, the molecular mechanism and the physiological effects underlying the incompatibility have remained enigmatic. In addition, it was unclear, if the ClpP protease is involved in the phenotype of the incompatibility.

## Results

### AB-I plants are unable to acclimate to higher light conditions

**Figure 2:**
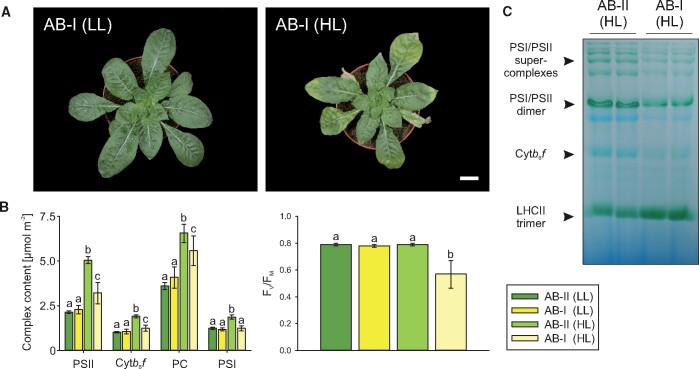
Light-dependent phenotype and physiology of AB-I plants. A, Yellow-green (*lutescent*) leaf phenotype and growth retardation under HL conditions (right). Plants are pictured at the end of the early rosette stage ∼30 days after germination. All molecular genetic and physiological analyses presented in this work were performed at this developmental stage. Bar = 5 cm. B, Analysis of photosynthetic parameters and photooxidative damage. (Left) Quantification of the components of the photosynthetic electron transport chain by difference absorbance spectroscopy. Note that AB-I plants under HL condition are not able to perform a typical light acclimation response by strongly increasing the content of all redox-active components of the electron transport chain relative to LL conditions. Bars represent mean values ± sd (*n* = 6–8 plants grown alongside). Right: Severe photooxidative damage of AB-I plants under HL conditions, exemplified by measurement of the maximum quantum efficiency of PSII in the dark-adapted state (*F*_V_/*F*_M_). Bars represent mean values ± sd (*n* = 6–8 plants grown alongside). Different lower case letters indicate significant differences (*P* < 0.05) according to two-way ANOVA with interactions followed by Tukey’s post-hoc testing (Supplemental File S4). C, Blue-native PAGE independently confirming the reduction of the electron transport chain complexes in AB-I under HL. Protein extracts equivalent to 30 μg chlorophyll were loaded in each lane. This experiment was performed two times independently with similar results.

AB-I plants display a yellow-green (*lutescent*) leaf chlorosis, caused by disturbed PSII activity (Greiner et al., 2008a; Figure 2, A and B). These photosynthetic defects occur specifically under high light (HL) intensities (Figure 2B). At 300 µE m^−2^s^−1^ (low light [LL]), the compatible wild-type AB-II and the incompatible hybrid AB-I are indistinguishable from each other, but higher light intensities cause severe photodamage in AB-I. Consistent with our previous study, already at 450 µE m^−2^s^−1^ (HL), a substantial portion of PSII was photodamaged. Interestingly, AB-I plants are also unable to perform an efficient light acclimation response when shifted to HL conditions (Figure 2B; see Supplemental Text in the Supplemental Data pdf file containing the Supplemental Figures and Supplemental Tables): Whereas AB-II plants responded to the increased growth light intensity by strongly increasing their chlorophyll content (Supplemental Figure S1A) and the contents of all redox-active components of the electron transport chain (Figure 2B), AB-I plants were incapable of performing this light acclimation response efficiently. This behavior, inhibited in AB-I plants, is a typical reaction when plants that were previously grown under light-limited conditions are transferred to higher light intensities (Schöttler and Tóth, 2014). Finally, this leads to a relative reduction of the components of the electron transport chain, namely PSI, PSII, and Cyt*b*_6_*f*, but not the ATP synthase and plastocyanin, in AB-I plants compared to AB-II (Figure 2, B and C; Supplemental Figure S1; Supplemental Text). In summary, AB-I plants display a light-dependent phenotype of photosynthetic acclimation that cannot be assigned to a single component of the electron transport chain. In addition, the disturbance in acclimation response is independent of ATP synthase and PC function.

### The better adaptation of wild-type AA-I plants to HL is conferred by the chloroplast genotype

To examine whether the genetic differences between plastomes I and II also have phenotypic effects under HL conditions in a compatible background (i.e. could be subject to selection in the parental species), we compared the light-acclimation response of green wild-type AA-I (*O. elata*) plants with green wild-type AB-II (*O. biennis*). In addition, to investigate whether potential differences are due to the chloroplast genome, we included the green chloroplast substitution line of the two genotypes (AA-II; Figure 1B). Since this experiment involved green material only, a harsher and more natural light shift from 300 to 600 µE m^−2^s^−1^ (harsh HL [HHL]) could be analyzed. The latter condition already induces severe damage in the incompatible AB-I genotype (Supplemental Text). However, it should be emphasized that 600 µE m^−2^s^−1^ is still a relatively moderate light intensity when compared to 1,000 to 1,500 µE m^−2^s^−1^ that can be reached in open land at noon.

In 300 µE m^−2^s^−1^, all photosynthetic parameters investigated (chlorophyll *a/b* ratio, chlorophyll content, *F_V_/F_M_*, linear electron transport capacity, and chloroplast ATP synthase activity) were very similar among the three genotypes (AA-I, AA-II, and AB-II; Table 1). However, after the shift to HL, pronounced differences were observed. While most parameters were not, or only weakly, affected in AA-I plants, the AB-II genotype showed a drastic loss of electron transport capacity. This was accompanied by marked decreases in the chlorophyll *a/b* ratio, chlorophyll content per leaf area, and chloroplast ATP synthase activity. Strikingly, similar changes also occurred in AA-II plants, indicating that the genetic background of the plastome, and not the nuclear genome, causes these differences in light acclimation. However, in comparison to typical light-acclimation responses of angiosperms, which (due to degradation of the chlorophyll *b* binding antenna proteins) result in increased electron transport capacity, chlorophyll content, and chlorophyll *a/b* ratio (Schöttler and Tóth, 2014), the response of AA-I plants to increased light intensity is limited. It, therefore, can be concluded that plastome I is better adapted to cope with HL conditions than plastome II, although at least under the conditions tested, the johansen Standard strain of *O. elata*, originally isolated in California (Cleland, 1935; Dietrich et al., 1997), does not behave like a typical HL plant.

**Table 1:** Comparison of light-acclimation responses of *O. elata* (AA-I), *O. biennis* (AB-II), and their green chloroplast substitution line AA-II

Parameter	AB-II 300 µE m^−2^ s^−1^	AB-II 600 µE m^−2^ s^−1^	AA-I 300 µE m^−2^ s^−1^	AA-I 600 µE m^−2^ s^−1^	AA-II 300 µE m^−2^ s^−1^	AA-II 600 µE m^−2^ s^−1^
Chlorophyll *a/b*	4.01 ± 0.06^a^	3.60 ± 0.15^b^	4.08 ± 0.05^a^	4.04 ± 0.16^a^	3.92 ± 0.08^a^	3.74 ± 0.15^b^
Chl. (mg m^−2^)	631.0 ± 18.1^ab^	548.6 ± 104.3^c^	668.1 ± 27.1^ad^	688.3 ± 58.8^d^	556.7 ± 8.4^b^	464.4 ± 34.1^e^
F_V_/F_M_	0.79 ± 0.01^a^	0.73 ± 0.08^b^	0.81 ± 0.01^a^	0.74 ± 0.06^b^	0.81 ± 0.01^a^	0.71 ± 0.03^b^
ETRII (µmol m^−2^ s^−1^)	172.3 ± 14.0^a^	75.1 ± 30.1^b^	171.5 ± 5.9^a^	137.5 ± 51.4^c^	147.9 ± 13.7^a^	84.4 ± 14.7^b^
gH^+^ (s^−1^)	39.2 ± 2.5^a^	27.4 ± 3.8^b^	43.0 ± 2.9^ac^	45.8 ± 11.0^c^	40.4 ± 3.6^a^	33.3 ± 2.8^b^

Plants were cultivated at either 300 µE m^−2^ s^−1^ or 600 µE m^−2^ s^−1^ actinic light intensity. Values are means ± SD (*n* = 5–6 plants grown in parallel). Different lowercase letters indicate significant differences (*P* < 0.05) according to two-way ANOVA with interactions followed by Tukey’s post-hoc testing (Supplemental File S4).

### RNA editing is not involved in the AB-I incompatibility

Chloroplast loci that cause the described phenotypes could be related to mRNA editing sites, which often display great variability among even closely related species (Schmitz-Linneweber et al., 2005; Kahlau et al., 2006). RNA editing in chloroplasts of seed plants involves C-to-U conversions at highly specific sites (Takenaka et al., 2013). It is of particular interest, since the only previously described mechanism of PGI is based on an editing deficiency of the tobacco (*Nicotiana tabacum*) *atpA* transcript (encoding a core subunit of the plastid ATP synthase) when exposed to the nuclear genetic background of the deadly nightshade *Atropa belladonna* (Schmitz-Linneweber et al., 2005). However, as evidenced by our sequencing results of the chloroplast transcriptomes of *O. elata* (AA-I), *O. biennis* (AB-II), and *O. grandiflora* (BB-III), mRNA editing does not play a role in the AB-I incompatibility. As judged from our data, all compatible wild-type genome combinations of the three species share the same 45 mRNA editing sites (Supplemental Table S2 and updated GenBank records AJ271079.4, EU262889.2, and KX014625.1). This analysis also includes partially edited sites, whose biological relevance is doubtful (see Supplemental Table S2 and “Methods” for details). These results exclude the possibility that editing sites in the plastome and/or nucleus-encoded editing factors differ among the genotypes involved in the AB-I incompatibility, as this was reported for an experimentally produced cytoplasmic hybrid (cybrid) of *Atropa* and tobacco (Schmitz-Linneweber et al., 2005).

### Association mapping to identify plastid loci causing the AB-I incompatibility

Having ruled out the involvement of mRNA editing, we performed an association mapping in the chloroplast genome of *Oenothera* to pinpoint the causative loci for the AB-I incompatibility. In contrast to the green alga *Chlamydomonas*, chloroplast genomes of higher plants are not amenable to linkage mapping (Chiu and Sears, 1985; Greiner et al., 2015). Hence, identification of functionally relevant loci is usually based on correlation of a polymorphism to a phenotype (e.g. Greiner et al., 2008a; Simon et al., 2016; Sobanski et al., 2019). In the case of the AB-I incompatibility, this can be achieved by manual inspection of an alignment of fully sequenced chloroplast genomes and a search for specific polymorphisms in plastomes I versus II, III, and IV. Those polymorphisms are considered candidates for causing the AB-I incompatibility because only plastome I confers the bleached *lutescent* phenotype in the AB nuclear genetic background, whereas plastomes II, III, and IV are all green when combined with the same nuclear genome (Stubbe, 1964; Greiner et al., 2008a). Our original analyses of the AB-I phenotype had included only 4 chloroplast genomes and yielded 16 candidate regions (Greiner et al., 2008a).

Using the power of next-generation sequencing (NGS) technologies, we were able to base the association mapping on 46 full chloroplast genomes whose genetic behavior had been determined by extensive crossing studies (Stubbe, 1959, 1960, 1963; Wasmund, 1980) (“Methods”; Supplemental Table S1). The chosen strains represent the material used for generalization of the genetic species concept in *Oenothera* that is based on the basic A, B, and C nuclear and I–V chloroplast genotypes (Cleland, 1972; Dietrich et al., 1997) (see “Introduction”). The strains reflect the full natural distribution range of *Oenothera* and cover all species accepted at the time the crossing data were gathered. Altogether, the mapping panel included 18 chloroplast genomes representing plastome type I (“Methods”; Supplemental Table S1). Although the individual plastomes differ by various single-nucleotide polymorphisms and insertions/deletions (indels) (cf. Greiner et al., 2008a, 2008b), only four polymorphisms were absolutely linked with the AB-I phenotype, that is, they were specific to plastome I and could potentially be involved in the AB-I incompatibility: (1) a 144-bp deletion in the *clpP*–*psbB* operon spacer region, (2) a combined 5-bp deletion/21-bp insertion in the *psbM*–*petN* spacer (genes encoding a PSII and a Cyt*b*_6_*f* subunit, respectively), (3) a 194 bp deletion in the *ndhG*–*ndhI* spacer (two genes encoding subunits of NADH dehydrogenase complex), and (4) a 21-bp insertion in the *trnL-UAA*–*trnT-UGU* spacer (Supplemental Files S1–S3).

Due to the lack of measurable sexual recombination frequencies in chloroplast genomes of seed plants (Greiner et al., 2015) (see above), genetic methods cannot be employed to further narrow down the causative loci for the AB-I incompatibility in plastome I. We, therefore, evaluated the remaining candidate polymorphisms with respect to their potential for causing the incompatible phenotype. Based on the deletions in the *ndhG*–*ndhI* and *trnL-UAA*–*trnT-UGU* spacers it was difficult to explain the observed light-dependent reduction of specific photosynthetic complexes in AB-I incompatible material (Figure 2). The neighboring genes do not encode components of the electron transport chain, and, moreover, knockouts of NDH complex subunits lack a discernible phenotype (Burrows et al., 1998). From possible effects on the expression of *trnL-UAA* and/or *trnT-UGU*, two essential tRNAs, one might expect a more pleiotropic phenotype that does not depend on the light intensity. As judged from the functions of the genes affected, a contribution of the latter two polymorphisms to the AB-I phenotype is hence unlikely, although strictly speaking we cannot fully exclude this. In contrast, the polymorphisms affecting the *psbB* operon and the *psbM*/*petN* spacer are more likely candidates because they can potentially affect both PSII and Cyt*b*_6_*f*, in agreement with the physiological data (Figure 1B).

### The *psbN*–*petN* spacer region makes a minor contribution to the AB-I incompatibility

**Figure 3:**
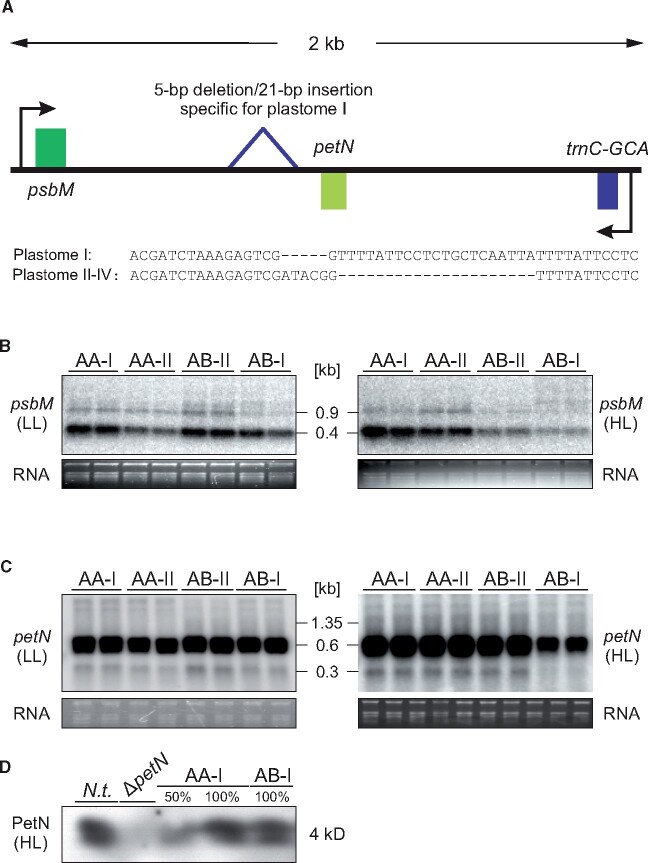
Molecular genetic analyses of the *psbM*–*petN* spacer region in compatible (AA-I, AA-II, and AB-II) and incompatible (AB-I) material under HL and LL conditions. A, Sequence context and indels (blue triangle) in the spacer that is specific to plastome I. Arrows indicate transcription start sites. B, C, RNA gel blot analyses of *psbM* (B) and *petN* (C) transcript accumulation under LL and HL conditions. These experiments were performed three times with similar results. D, Immunoblot analysis of PetN accumulation under HL. *N.t.* = *N. tabacum* wild type, Δ*petN* = *petN* knockout in *N. tabacum* (Hager et al., 1999). 100% corresponds to 5 µg chlorophyll equivalent. Tobacco control lines were grown in tissue culture as described in “Methods.” The experiment was performed independently three times with similar results.

To examine the contribution of the combined 5-bp/21-bp indel in the *psbM*–*petN* spacer, transcript and protein analyses were performed in incompatible AB-I plants and compatible controls under LL and HL conditions (Figure 3). The indel is located in the 3'-UTR of both genes (Figure 3A) and, therefore, could potentially affect the stability of their transcripts.

RNA blot analyses revealed that both genes are affected by the indel. For *psbM*, reduction of the 0.35-kb monocistronic transcript was observed for AA-II and AB-I under LL, and for AB-II and AB-I under HL conditions. Although there is no obvious explanation for this light-dependent effect, it is independent of the AB-I incompatibility because the levels of *psbM* mRNA cannot be linked to the AB-I phenotype (Figure 3B and below). Moreover, as judged from knockout mutants in tobacco, even complete loss of the PsbM protein does not lead to a strong phenotype that would be comparable to the phenotype of AB-I plants (Umate et al., 2007). In contrast, *petN* encodes an essential subunit of the Cyt*b*_6_*f* (Hager et al., 1999; Schwenkert et al., 2007), and reduced *petN* transcript stability, therefore, could affect Cyt*b*_6_*f* accumulation. RNA gel blot analysis of *petN* mRNA accumulation detected a mature transcript of 0.3 kb (Figure 3A). Under LL conditions, *petN* transcript accumulation is unaltered in the incompatible hybrid, whereas under HL, the *petN* mRNA is significantly reduced in AB-I material (Figure 3C). Immunoblot analyses showed that this leads to a reduction of the protein level to ∼80% (Figure 3D), an estimate that is well supported by our spectroscopic quantification of Cyt*b*_6_*f* (Figure 1B).

Taken together, these data do not exclude the possibility that the *psbM*/*petN* region influences the incompatibility phenotype, but suggest a rather minor contribution. However, involvement of *psbM* is unlikely, because down-regulation of its mature transcript is observed also in compatible AA-II plants under LL conditions (Figure 3B). Nonetheless, a role of *petN* is unlikely as well, since reduction of ∼20% of the Cyt*b*_6_*f* content (Figures 2B and 3D) does not affect accumulation of the PSs (Anderson et al., 1997; Hager et al., 1999; Schwenkert et al., 2007; Schöttler et al., 2007b). Hence, another chloroplast locus is responsible for the AB-I incompatibility.

### The promotor region of the *psbB* operon is a major determinant of the AB-I incompatibility

Next, we analyzed the transcript patterns of the *clpP* and *psbB* operons that flank the 144-bp deletion in the spacer region (Supplemental Figure S2A). RNA gel blot analyses revealed that accumulation of both the *clpP* precursor transcript and the mature *clpP* mRNA did not differ in control plants versus incompatible plants under HL conditions. All *clpP* transcripts accumulated to similar levels as in the compatible lines. Similarly, no difference in transcript accumulation of the remaining operon genes residing upstream of *clpP* (*rpl20* and *5'-rps12*) was observed (Supplemental Figure S2B). In addition, analyses of ClpP protein accumulation and the integrity of the plastid ribosomes revealed no difference between compatible and incompatible material (Zupok, 2015). Based on these findings, a contribution of the *clpP* operon to the incompatibility phenotype can be excluded.

**Figure 4:**
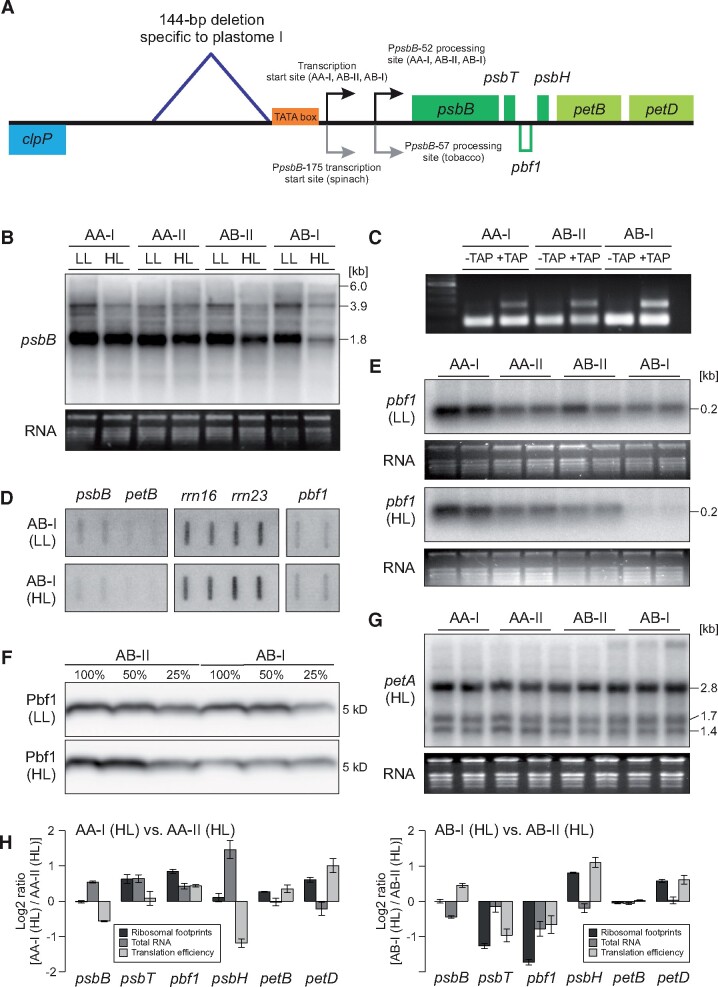
Regulation of the *psbB* operon in compatible (AA-I, AA-II, and AB-II) and incompatible (AB-I) plants under HL and LL conditions. A, Physical map of the region in the chloroplast genome containing the *clpP* and *psbB* operons. The 144-bp deletion in the intergenic spacer upstream of the *psbB* operon promoter is indicated. Transcription start sites and mRNA processing sites are indicated by arrows. Note that *pbf1* (encoded on the opposite strand) is transcribed from its own promoter. B, RNA gel blot analysis of *psbB* transcript (representative of the whole *psbB* operon; also see Supplemental Figure S2). C, 5'-RACE with and without TAP treatment, a method to map transcription start sites and RNA processing sites of the *psbB* operon. For details, see “Methods.” D, Run-on transcription analysis of the *psbB* operon (*psbB* and *petB*), including appropriate controls (*rrn16*, *rrn23*), and *pbf1*. E, RNA gel blot analysis of *pbf1* transcripts. F, Immunoblot analysis of Pbf1 accumulation. 100% corresponds to 3 µg chlorophyll equivalent. G, RNA gel blot analysis of *petA* transcript accumulation, serving as a control for a gene outside of the *psbB* operon. H, Ribosomal profiling and translation efficiency of the *psbB* operon of AA and AB genotypes under HL conditions. Note a tendency of increased translation in AA-I versus AA-II (left). In turn, upon presence of the B genome, translation of *psbT* and *pbf1* is reduced in incompatible AB-I plants when compared to compatible AB-II (right). B–G, With the exception of (F) all experiments were performed independently three times with similar results. F, was performed two times with similar results. H, The experiment is based on three replicates from a pool of ∼20 individuals per genotype. Bars represent mean values ± sd.

In contrast, transcript accumulation of all *psbB* operon genes (*psbB*, *psbT*, *psbH*, *petB*, and *petD*) was found to be reduced in AB-I plants under HL, but not LL conditions (Figure 4, B and F; Supplemental Figure S2). Run-on transcription analyses revealed that this effect was due to impaired transcription rather than being an effect of altered transcript stability. Transcription of the *psbB* operon was specifically reduced under HL conditions in the incompatible hybrids (Figure 4D). Consequently, in contrast to the green AA-I, AA-II, and AB-II plants, the deletion in plastome I in the AB background affected regulation of the *psbB* operon promoter in a light-dependent manner (Figure 4D). Importantly, the same promoter was used in all genetic backgrounds, as evidenced by mapping of the transcription start sites (Figure 4C), which are also highly conserved among species (Figure 4A). The deletion does not affect the TATA box of the *psbB* operon promoter, but resides 7-bp upstream of the −35 box. This may suggest that polymerase binding is not affected, but instead that binding of auxiliary proteins such as sigma factors is impaired by the deletion in the incompatible hybrids (see “Discussion”).

Interestingly, *photosystem biogenesis factor 1* (*pbf1*, previously designated *psbN*), a gene involved in PSI and PSII assembly (Krech et al., 2013), is downregulated in AB-I incompatible plants under HL conditions (Figure 4E). *pbf1* is transcribed from the opposite strand by its own promoter, which lacks any polymorphism in all *Oenothera* plastomes sequenced so far (Supplemental Files S1–S3). Therefore, the reduction in *pbf1* transcript accumulation might result from the sense-antisense interaction with the *psbT* mRNA, as previously described for *Arabidopsis* (Zghidi-Abouzid et al., 2011; Chevalier et al., 2015). Alternatively, it could be the result of a type of unknown feedback regulation. In any case, the interaction results in a strong reduction of Pbf1 accumulation (Figure 4F) that is a result of a decreased translation activity of the reduced *pbf1* transcript and its antisense counterpart *psbT* (Figure 4H). Since *pbf1* knockouts are extremely light-sensitive and show severe defects in PSII and, to a lesser extent, also in PSI accumulation (Krech et al., 2013; Torabi et al., 2014), it appears likely that the effect on the *pbf1* mRNA also contributes to the incompatibility phenotype.

## Discussion

PGI was reported from a large number of plant species and is an important phenomenon in plant speciation (Greiner et al., 2011). Our work reported here shows that light-dependent misregulation of a core photosynthesis operon leads to hybrid incompatibility, thus causing reproductive isolation and ultimately, speciation. Interestingly, the underlying genetic architecture was shaped during the last ice age by periods of glaciation, as noted in the Introduction. The mechanism we have discovered is different from that of the two other cases of PGI mechanistically studied so far. RNA editing of the *atpA* transcript was identified as the cause of chloroplast–nuclear incompatibly in an *Atropa*/tobacco synthetic cybrid (Schmitz-Linneweber et al., 2005) (see above). Variation in the coding regions of *accD* (the plastid-encoded subunit of the acetyl-CoA carboxylase, catalyzing the first step of fatty acid biosynthesis) was suggested as a genetic determinant of PGI in pea (Bogdanova et al., 2015). However, the *Atropa*/tobacco case represents an unnatural, artificial combination of the plastid and the nuclear genomes of two sexually incompatible species, and, unfortunately, the evidence for possible causative loci for the incompatibility in pea are currently not strong enough to judge their impact on natural populations (Nováková et al., 2019). Moreover, in both cases, the ecological relevance of the suggested PGI loci is unclear and cannot be deduced from the identified polymorphisms.

In contrast, our data indicate that the AB-I incompatibility might have evolved as a result of ecological selection, representing a genotype × genotype × environment interaction (G×G×E). Although cytoplasmic G×E interactions were reported earlier in bread wheat (*Triticum aestivum*; Ekiz et al., 1998), especially for the chloroplast, cases in plants are hardly described. Recent work in animals, however, indicates that G×G×E interactions may be relatively common (Hoekstra et al., 2018; Hill et al., 2019; Rand and Mossman, 2020). That cytoplasmic incompatibly, in turn, can result from ecological selection is obvious from work in sunflower (using a cross between *Helianthus annuus* and *H. petiolaris*), in which common garden experiments in xeric and mesic habitats demonstrated maintenance of CI by positive selection (Sambatti et al., 2008). The underlying genes and physiology, however, have remained enigmatic.

Our study demonstrates that photosynthesis-related genes encoded in the chloroplast genome can establish hybridization barriers. The incompatible phenotype is only visible under HL condition—AB-I plants cannot perform the necessary acclimation response as described above. Strikingly, plastome I in the native AA nuclear genetic background of *O. elata* (a species adapted to the western United States and Mexico) copes better with HL conditions than does the AB-II genotype of *O. biennis*, which is native to the North American woodlands (Dietrich et al., 1997). This effect is plastome-dependent, since the green AA-II chloroplast substitution line behaves photosynthetically very similar to *O. biennis*, but fully resembles the *O. elata* morphotype. However, to what extent the identified incompatibility locus is involved in a light acclimation response of AA-I species in their natural habitats remains to be addressed in further investigations.

Within their natural distribution ranges, species carrying plastome I colonized central and southwest North America (xeric habitats with HL irradiation), whereas species carrying plastomes II, III, IV, or V are limited to the mesic sites of eastern North America (Figure 1C) (Cleland, 1972; Dietrich et al., 1997). Hence, plastome I seems to be required for colonization of habitats exposed to higher irradiation. This assumption is further supported by the fact that *O. biennis* (AB-II or BA-III), a species that spread west of the Great Plains after 1970, is only rarely found in the southern parts of the United States and is still absent from Mexico (Cleland, 1972; Dietrich et al., 1997).

Consequently, the loci underlying the AB-I incompatibility seem to prevent colonization of the south western parts of North America by the B genome by creating an asymmetric hybridization barrier between AA-I and AB-II, BA-III, and BB-III species. At the same time, the compatible nuclear–chloroplast genome combination AA-I may have facilitated physiological adaptation of the corresponding species by nuclear–cytoplasmic coevolution. Therefore, it seems reasonable to assume that, as a result of higher light intensities (and/or light quality differences) in xeric habitats, the deletion upstream of the *psbB* operon promoter coevolved with nucleus-encoded proteins that interact with the (bacterial-type) plastid-encoded RNA polymerase (PEP).

Strong candidates for these interacting proteins are the PEP sigma factors, which were shown to regulate polymerase binding in response to both light quality and light quantity (Noordally et al., 2013; Chi et al., 2015). Moreover, regulation by sigma factors (e.g. through redox-induced phosphorylation) influences the stoichiometry of the protein complexes of the photosynthetic electron transport chain (Shimizu et al., 2010). Thus, the failure of AB-I plants to acclimate to HL intensities could be a direct consequence of disturbed transcriptional regulation by sigma factors. Coevolution and coordinated rates of molecular evolution of PEP core subunits and sigma factors could be a common principle in plant evolution, as suggested by recent findings in Geraniaceae, a family in which PGI is also widespread (Zhang et al., 2015).

Finally, it should be emphasized that, although the *psbB* operon does not encode PSI-related genes, its transcriptional misregulation also explains the observed effect on PSI, due to either the antisense interaction with the *pbf1* mRNA or an unknown mechanism of feedback regulation (Figure 4). Interestingly, while the *psbB* operon (including the *pbf1* gene on the opposite strand) displays extremely high structural conservation from cyanobacteria to higher plants, light regulation of *pbf1* transcript abundance was shown to be highly variable among species (Plöchinger et al., 2016).

## Materials and methods

### Plant material

Throughout this work, the terms *Oenothera* and evening primrose refer to subsection *Oenothera* (genus *Oenothera*, section *Oenothera*, Onagraceae; 2*n* = 2*x* = 14) (Dietrich et al., 1997). Plant material used here is derived from the *Oenothera* germplasm resource harbored at the Max Planck Institute of Molecular Plant Physiology (Potsdam-Golm, Germany). This includes the living taxonomic reference collection of subsection *Oenothera* (Greiner and Köhl, 2014). Part of this reference collection is the Renner Assortment, a medium-sized collection of European lines that were thoroughly characterized by members of the genetic school of Otto Renner (Cleland, 1972; Harte, 1994). In addition, it includes the Cleland collection, a large set of North American strains of subsection *Oenothera* that was extensively studied by Ralph E. Celand and colleagues (Cleland 1972). Also present are North American accessions analyzed by Wilfried Stubbe et al., and these represent species of this subsection that were recognized later than the 1960s (Stubbe and Raven, 1979a; Steiner and Stubbe, 1984, 1986; Wasmund and Stubbe, 1986; Wasmund, 1990; Schumacher et al., 1992; Schumacher and Steiner, 1993; Stubbe and Steiner, 1999). The availability of this material allowed us to employ the original source of lines on which the genetic species concept of subsection *Oenothera* was based (cf. Cleland, 1962) The lines employed for association mapping of the plastidic AB-I locus were extensively analyzed by classical genetics for the compatibility relations of their nuclear and chloroplast genomes (Supplemental Table S3 for details).

RNA editing analyses were performed with the wild-type strains of *O. elata* subsp. *hookeri* strain johansen Standard (AA-I), *O. grandiflora* strain Tuscaloosa (BB-III), and *O. biennis* strain suaveolens Grado (AB-II). Supplemental Table S3 contains a summary of all wild-type strains used in this work.

For most other genetic or physiological work presented here, the wild-type strains johansen Standard (AA-I) and suaveolens Grado (AB-II), or chloroplast substitution lines between them (AA-II and AB-I) were used. Here, AA-I refers to the wild-type situation, that is, strain johansen Standard with its native nuclear and chloroplast genomes. AA-II refers to the nuclear genome of johansen Standard combined with the chloroplast genome of suaveolens Grado. AB-II designates nuclear and chloroplast genomes of the wild-type strain suaveolens Grado, and AB-I the nuclear genome of suaveolens Grado equipped with the chloroplast genome of johansen Standard. Generation of AA-II and AB-I from the wild-types AB-II and AA-I is detailed below (see the “Summary” in Supplemental Table S4).

For ribosomal profiling and RNA-seq analyses incompatible (AB-I) and compatible (AB-II) plants were obtained from F1 seeds of crosses between the wild-type or mentioned chloroplast substitution lines of johansen Standard (AA-I or AA-II) as the seed parent and grandiflora Tuscaloosa (BB-III) as the pollen parent. Here, AA-I × BB-III yielded AB-I plants, and AA-II × BB-III produced AB-II plants. Selection against the paternally transmitted plastome III was done using appropriate markers (Rauwolf et al., 2008). The material produced largely recreates the AB-I genotype that was characterized by Greiner et al. (2008a). For the tobacco (*N. tabacum*) wild-type, the cultivar Petit Havana was used. The tobacco Δ*petN* mutant was obtained from Hager et al. (1999).

### Generation of chloroplast substitution lines

In *Oenothera*, the genetics of permanent translocation heterozygosity, combined with a biparental transmission of plastids, offer an elegant opportunity to substitute chloroplasts between species in only two generations while leaving the nuclear genome constitution unaltered (Stubbe, 1959, 1960, 1989). For the interested reader, general principles, including a detailed discussion of crossing examples, are presented in Rauwolf et al. (2008). The chloroplast substitution between the strains suaveolens Grado and grandiflora Tuscaloosa (described in Figure 6 of Rauwolf et al. 2008) resembles the chloroplast substitution between suaveolens Grado and johansen Standard used in this work.

In brief, due to reciprocal chromosomal translocations, many species of *Oenothera* form permanent multichromosomal meiotic rings. If all members of a given chromosome complement are involved in a single ring, they establish two regularly segregating sets of genetically linked chromosomes. This leads to formation of two superlinkage groups, each involving one complete parental haploid chromosome set (α and β). Suppression of homologous recombination avoids genetic reshuffling between the two haploid sets. Additional genetic properties, especially presence of gametophytic lethal factors that lead to sex-linked inheritance of a given haploid set, eliminate homozygous segregants (α·α or β·β). This results in permanent heterozygous progeny (α·β) that is identical to the parental plant. The phenomenon of structural heterozygosity is a form of functional asexuality. However, truly sexual species also exist in *Oenothera*, that is, species that display bivalent-pairing and regular meiotic segregation. In contrast to the structurally heterozygous species, they lack lethal factors and are homozygous for their haploid sets (haplo·haplo versus α·β from above; Cleland, 1972; Harte, 1994).

As a consequence of this genetic behavior, entire haploid chromosome sets in evening primrose can behave as alleles of a single Mendelian locus. These so-called Renner complexes are designated using (Latin) names; for example, *^G^albicans*·*^G^flavens* (α·β) for the structurally heterozygous strain suaveolens Grado or *^h^johansen Standard*·*^h^johansen Standard* (haplo·haplo) for the homozygous line johansen Standard. A cross between them (suaveolens Grado × johansen Stanard = *^G^ablicans*·*^G^flavens* x *^h^johansen Standard*·*^h^johansen Standard*) yields in the F1 the offspring *^G^albicans*·*^h^johansen Standard* and *^G^flavens*·*^h^johansen Standard*.

To equip johansen Standard (AA-I) with the chloroplast of suaveolens Grado (AB-II), the F1 hybrid *^G^albicans*·*^h^johansen Standard* was used. (The other hybrid *^G^flavens⋅^h^johansen Standard* is not of interest and therefore discarded.) Due to biparental inheritance of chloroplasts in evening primroses, it carries the chloroplasts of both johansen Standard (I-johSt) and suaveolens Grado (II-suavG). (Note that nuclear genomes are indicated in italic and chloroplast genomes in nonitalic text.) Since *^G^albicans*·*^h^johansen Standard* I-johSt/II-suavG displays a full meiotic ring, this leads to suppression of homologous recombination as well as elimination of random chromosome assortment in meiosis (see above). Therefore, as a result of Mendelian segregation of the *^h^johansen Standard* complex, the johansen Standard strain (*^h^johansen Standard*·*^h^johansen Standard*) can be recreated from *^G^albicans*·*^h^johansen Standard* upon selfing. (*^G^albicans*·*^h^johansen Standard* × s = *^G^albicans*·*^G^albicans*, *^G^albicans*·*^h^johansen Standard* and *^h^johansen Standard*·*^h^johansen Standard*; the sergeant *^G^albicans*·*^G^albicans* is not realized due to a male gametophytic lethal factor in *^G^albicans*). When a *^G^albicans*·*^h^johansen Standard* plant homoplasmic for II-suavG is used for selfing, the johansen Standard plant in F2 now carries plastome II-suavG (AA-II). If AB-I plants are desired, the *^G^albicans*·*^h^johansen Standard* I-johSt/II-suavG hybrid in F1 is selected for I-johSt and backcrossed with suaveolens Grado (*^G^abicans*·*^G^flavens* II-suavG). BC1 then reassembles *^G^albicans*·*^G^flavens* I-johSt/II-suavG which, due to the maternal dominance of biparental transmission in evening primrose (Schötz, 1954; Chiu et al., 1988; Sobanski et al., 2019), contains a major proportion of *^G^albicans*·*^G^flavens* I-johSt, that is, AB-I plants.

### Plant cultivation, growth conditions, and tissue harvest

For crossing studies, plastome sequencing, and analysis of RNA editing, *Oenothera* plants were cultivated in a glasshouse as previously described (Greiner and Köhl, 2014). AA-I, AA-II, AB-I, and AB-II plants for molecular genetics and physiological analyses were cultivated in soil in growth chambers using a 16-h light/8-h darkness cycle and 24°C at LL intensities (∼150 µE m^−2^s^−1^). At the beginning of the early rosette stage, ∼21 days after germination (cf. Greiner and Köhl, 2014), plants were transferred to higher light intensities, that is, 300 µE m^−2^s^−1^ (LL), 450 µE m^−2^s^−1^ (HL), or 600 µE m^−2^s^−1^ (HHL), and then kept under the same growth regime. Hence, in all subsequent experiments the material was analyzed during the early rosette stage, which is defined as the young rosette 21–30 days after germination (see Greiner and Köhl [2014] for details and Figure 1A). About 600 µE m^−2^s^−1^ was used only for a single experiment, because it resulted in severe photodamage of the incompatible combination AB-I (see Supplemental Text). To avoid pleiotropic effects, the yellowish material of the bleached leaf tip, a typical characteristic of the *lutescent* AB-I incompatible phenotype (Figure 1A), was excluded from all experiments. The tobacco Δ*petN* mutant and its corresponding wild-type were cultivated as reported earlier (Hager et al., 1999).

### Thylakoid membrane isolation from *Oenothera* leaves

For spectroscopic measurements and blue native–PAGE, an improved thylakoid membrane isolation protocol was developed for *Oenothera* leaf tissue that contains high amounts of mucilage and starch. All steps were performed at 4°C. Solutions were pre-chilled, leaves shortly placed in ice-cold water (H_2_O), and dried with a salad spinner. Approximately 10 g of mature leaf tissue that was dark adapted for 1 h was homogenized in a blender adding 200 mL of isolation buffer (330 mM sorbitol, 50 mM HEPES, 25 mM boric acid, 10 mM EGTA, 1 mM MgCl_2_, 10 mM NaF [optional, if protein degradation is apprehend]; pH 7.6 with KOH, and 5 mM freshly added sodium ascorbate). Then 100-mL aliquots of the homogenate were then filtered through a double layer of cheese cloth (Hartmann, Heidenheim, Germany), followed by filtering through a single layer of Miracloth (Merck, Kenilworth, NJ, USA). After that, the following procedure was applied twice: after adjustment of the solution to 200 mL with isolation buffer, it was centrifuged for 5 min at 5,000 *g* and the pellet was resuspended in 40 mL of isolation buffer using a 30-cm^3^ Potter homogenizer (VWR, Radnor, PA, USA; mill chamber tolerance: 0.15–0.25 mm). Following the second homogenization step, the solution was adjusted to 200 mL with washing buffer (50 mM HEPES/KOH [pH 7.6], 5 mM sorbitol, and optionally 10 mM NaF) followed by a filtering step through one layer of Miracloth. Subsequently, the thylakoid homogenate was centrifuged for 5 min at 5,000 *g*, the pellet resuspended with a 30-cm^3^ Potter homogenizer in 30 mL washing buffer and centrifuged for 5 min at 5,000 *g*. Then, after resuspending the thylakoids in 5 mL of washing buffer, the homogenate was placed on a 85% Percoll cushion (Percoll stock solution: 3% [w/v] polyethylene glycol 6,000, 1% [w/v] BSA, 1% [w/v] Ficoll 400, dissolved in Percoll; 85% Percoll: 85% PBF-Percoll stock solution, 330 mM sorbitol, 50 mM HEPES, 2 mM EDTA, 1 mM MgCl_2_; pH 7.6 with KOH) in a 30 mL Corex tube and centrifuged for 5 min at 5,000 *g*. This step effectively removes starch from the isolation. Finally, thylakoids, which do not enter the Percoll cushion, are collected, washed in a total of 25 mL of washing buffer, centrifuged for 5 min at 5,000 *g*, and resuspended in the desired buffer and volume.

### Spectroscopic methods

For quantification of isolated thylakoids, chlorophyll amounts were determined in 80% (v/v) acetone (Porra et al., 1989). The contents of PSII, PSI, Cyt*b_6_f*, and PC were determined in thylakoids as described previously (Schöttler et al., 2004). PSI was quantified from P700 difference absorption signals at 830–870 nm in solubilized thylakoids using the Dual-PAM-100 instrument (Walz, Effeltrich, Germany) (Schöttler et al., 2007a, 2007b). Contents of PSII and Cyt*b_6_f* were determined from difference absorption measurements of cytochrome b_559_ and Cyt*b_6_f*, respectively. Measurement procedures and data deconvolution methods have been described previously in detail (Kirchhoff et al., 2002; Schöttler et al., 2007a). Maximum *F_v_/F_m_* values were measured in leaves adapted to darkness for 1 h. Chlorophyll fluorescence was recorded with a pulse amplitude-modulated fluorimeter (Dual-PAM-100) on intact plants at room temperature. An F-6500 fluorometer (Jasco, Tokyo, Japan) was used to measure 77 K chlorophyl-*a* fluorescence emission spectra using an amount of freshly isolated thylakoid membranes equivalent to 10 μg chlorophyll mL^−1^. The sample was excited at 430 nm (bandwidth of 10 nm), and the emission spectrum was recorded between 655 and 800 nm in 0.5 nm intervals (bandwidth of 1 nm). Dark-interval relaxation kinetics of the electrochromic shift, a measure of the proton motive force across the thylakoid membrane, was used to determine the thylakoid conductivity for protons (gH^+^), which is a proxy for ATP synthase activity. Electrochromic shift signals were measured and deconvoluted using a KLAS-100 spectrophotometer (Walz) as previously described (Rott et al., 2011).

### Antibody sources and anti-PetN serum production

The anti-Pbf1 (PsbN) antibody used in this work was described in Torabi et al. (2014). The serum was diluted 1:1,000. The anti-AtpA (AS08 304; dilution 1:5,000) antibody and the secondary antibody (Goat anti-Rabbit IgG [H&L, Bristol, UK], HRP conjugated, AS09 602, dilution 1:40,000) were obtained from Agrisera (Vännäs, Sweden). To prepare an antibody against the PetN protein, rabbits were injected with 8-amino-3,6-dioxaoctanoic acid (PEG2)-FTFSLSLVVWGRSGL-PEG2-C-Amid (BioGenes GmbH, Berlin, Germany), a highly hydrophobic peptide comprising about half of the small protein PetN. The peptide was coated with PEG2 to ensure better solubility. Active serum was obtained after four immunizations and used in a dilution of 1:500.

### Protein analyses

Blue native–PAGE was performed as previously reported (Ossenbühl et al., 2004; Schwenkert et al., 2006). To avoid protein degradation, 10 mM of NaF was optionally added to all solutions. Thylakoid membranes were solubilized with dodecyl-β-d-maltoside at a final concentration of 1% and separated in 4%–12% polyacrylamide gradient gels. Protein equivalents of 30 μg chlorophyll were loaded in each lane.

For immunoblot analyses, thylakoids were mixed with sample buffer (50 mM Tris/HCl [pH 6.8], 30% [v/v] glycerol, 100 mM DTT, 4% [w/v] SDS, 10% [w/v] Coomassie Brilliant Blue G-250) and were denatured for 5 min at 95°C under continuous agitation. Then, samples were analyzed by Tricine–SDS–PAGE (16%T separation gel and 4%T stacking gel) followed by gel blotting onto a PVDF membrane (0.2 µm) using the semi-dry PEQLAB transfer system (PEQLAB Biotechnologie GmbH, Erlangen, Germany). After incubation with the secondary antibody, immunochemical detection was performed with the help of the ECL Prime Western Blotting Detection Reagent (GE Healthcare, Chicago, IL, USA) according to the supplier’s recommendations. In the relevant figures, 100% loading represents a 3 µg chlorophyll equivalent.

### Isolation and purification of nucleic acids

DNA and RNA isolations from evening primroses were performed employing protocols specially developed for their mucilage and phenolic compound rich tissue, as previously described in Massouh et al. (2016). For RNA-seq analyses, total RNA was purified from residual DNA contamination by digestion employing the Ambion^®^ Turbo DNA-free™ Kit (Thermo Fisher Scientific, Waltham, MA, USA).

### Association mapping of the plastid AB-I locus

For association mapping in the chloroplast genome, we used 46 full plastome sequences of *Oenothera* for which precise genetic information is available (Supplemental Table S1 and “Plant material” section). To this end, we newly determined the sequences of 30 plastomes, now available from GenBank under the accession numbers KT881175.1, KX014625.1, MN807266.1, MN807267.1, and MN812468.1–MN812493.1. The new chloroplast genomes were annotated and submitted by GeSeq version 1.43 (Tillich et al., 2017) and GB2sequin version 1.3 (Lehwark and Greiner, 2019), respectively. The remaining 16 plastomes were previously published (see Supplemental Table S1 for details). Chloroplast genome sequencing from *Oenothera* total DNA was done as reported earlier (Massouh et al., 2016; Sobanski et al., 2019), but a higher version of the SeqMan NGen assembly software was used (version 14.1.0; DNASTAR, Madison, WI, USA). Also, in contrast to earlier work, 250-bp Illumina paired-end reads (instead of 100-bp or 150-bp) were generated, with the exception of KX014625.1 (100-bp paired-end) and MN807266.1 and MN807267.1 (both 150-bp paired-end). Subsequently, for association mapping, the redundant inverted repeat A (IR_A_) was removed, sequences were aligned with ClustalW (Thompson et al., 1994) and the alignments manually curated in Mesquite version 3.61 (Maddison and Maddison, 2018). Polymorphisms specific to plastome I (i.e. polymorphisms that were present in all 18 plastome I genotypes but absent from all 28 plastomes II, III, and IV genotypes) were identified by visual inspection in SeqMan Pro version 15.2.0 (DNASTAR) (cf. Greiner et al., 2008a). For the original data file, see Supplemental File S2. For readers without access to the commercial SeqMan Pro software, Supplemental File S1 and S3 are provided. Those contain the multiple sequence alignment of the 46 plastomes in standard FASTA, as well as the annotation of its consensus in the widely used GenBank format, respectively. Both files together reassemble the information included in Supplemental File S2, but can be read by any (free) sequence analyses software.

### RNA editing analyses

To determine the RNA editotype of the *Oenothera* chloroplast, RNA-seq samples of the 1kp project (Hollister et al., 2015; Leebens-Mack et al., 2019) of johansen Standard (AA-I), suaveolens Grado (AB-II), and grandiflora Tuscaloosa (BB-III; NCBI SRA Accession Numbers ERS631151, ERS631122, and ERS631139; also see “Plant material” section) were mapped against their respective chloroplast genomes (AJ271079.4, KX014625.1, and EU262889.2) from which the IR_A_ had been removed. For this we employed the reference-guided assembly—special workflows pipeline of SeqMan NGen version 15.2.0. single-nucleotide polymorphisms were called in SeqMan Pro version 15.2.0. To deal with the heterogeneity of the mRNA population, partial editing, and sequencing errors, sites showing C-to-T (U) conversion of at least 30% were originally considered as mRNA editing sites. If editing could not be detected above this threshold at a given site in all three species, the sites were subjected to manual inspection of the original mapping data. In most cases, this procedure revealed mapping errors, however, in a few cases also partial editing ˂30% in at least one of the strains was uncovered.

### Gel blot detection of RNA

RNA blot analyses were performed as previously described (Massouh et al., 2016). Gene-specific PCR products used as probes were obtained by using the primers listed in Supplemental Table S5. Total *Oenothera* DNA was used as template in standard PCR reactions.

### Chloroplast run-on analyses

For slot-blot preparation of DNA probes, PCR-amplified DNA probes (Supplemental Table S5) were immobilized to a Hybond-N+ nylon membrane (Amersham, Little Chalfont, UK) through a slot-blot manifold. For this, 1.5 μg of DNA was denatured in 0.5 M NaOH and heated for 10 min at 95°C. Then, the volume of the denatured DNA probes was adjusted with H_2_O to 100 μL per spot. After heating, the probes were cooled on ice for 2 min to prevent DNA renaturation, and briefly centrifuged to collect the condensate. To each sample, 20 μL of cold 0.5 M NaOH and 0.5 μL of cold 10× DNA loading-dye (50% [v/v] glycerol, 100 mM EDTA, 0.25% [w/v] bromophenol blue, 0.25% [w/v] xylene cyanol) were added. Subsequently, the samples were spotted to nylon membranes pre-hydrated with double-distilled H_2_O, and then 100 μL 0.5 M NaOH was applied to each spot. After drying the membrane at room temperature for 5 min, the DNA was cross-linked to the membrane with 0.12 J/cm^2^ using the UV crosslinker BLX-254 (BIO-LINK, Liverpool, NY, USA).

To analyze strand-specific gene expression of *pbf1*, single-stranded *pbf1* RNA probes were generated using the Ambion^®^ Maxiscript^®^ T7 Kit (Invitrogen, Carlsbad, CA, USA) according to the manufacturer’s instructions and immobilized through a slot-blot manifold to a Hybond-N+ nylon membrane (Amersham). The *pbf1* gene of johansen Standard was amplified with the primer pair psbNRO_F 5′-AGCATTGGGAGGCTCATTAC-3′ and psbNRO_R 5′-GGAAACAGCAACCCTAGTCG-3′ and cloned into to pCRTM2.1-TOPO^®^ (Invitrogen). The vector was linearized with *Hind*III and in vitro transcription was performed according to the suppliers’ protocol. About 1.5 μg of RNA was adjusted with nuclease-free H_2_O to a volume of 50 μL prior to incubation with 30 μL of 20× SSC (0.3 M sodium citrate and 3.0 M sodium chloride) and 20 μL 37% formaldehyde at 60°C for 30 min. Samples were maintained on ice and spotted to nylon membranes pre-hydrated with double-distilled H_2_O and 10× SSC. Next, 100 μL 10× SSC was applied per slot. After drying the membrane at room temperature for 5 min the RNA was cross-linked with an UV crosslinker as described above.

For in vitro transcription and hybridization to slot-blot membranes, chloroplasts from *Oenothera* leaves harvested 8–10 weeks after germination were isolated and counted according to a previously published protocol, applying the same minor modifications as described in Sobanski et al. (2019). Then, a chloroplast suspension containing 4.9 × 10^7^ chloroplasts was transferred to a fresh tube, centrifuged at 5,000 *g* for 1 min and the supernatant was removed. To start the in vitro transcription, 20 units of RNase inhibitor (Promega GmbH, Madison, WI, USA), 50 μCi of (α-32P) UTP, and 94 μL transcription buffer (50 mM Tris/HCl [pH 8.0], 10 mM MgCl_2_, 0.2 mM CTP, GTP, and ATP, 0.01 mM UTP, 10 mM 2-mercaptoethanol) were added, mixed, and incubated for 10 min at 25°C. Next, the reaction was stopped by adding 10 μL of stop buffer (5% [w/v] Na-lauroylsarcosine, 50 mM Tris/HCl [pH 8.0], 25 mM EDTA) followed by a RNA isolation protocol, where 100 μL of phenol/chloroform/isoamyl alcohol (25:24:1) was added to the reaction, vortexed, incubated for 10 min at room temperature, and centrifuged at 18,000 *g* for 10 min at 4°C. Afterward, the upper phase was collected and nucleic acids were precipitated overnight at −20°C using 3 volumes of 100% (v/v) ethanol, 0.3 M sodium acetate, and 1 μL GlycoBlue™ (Invitrogen). On the next day, the sample was centrifuged at 20,000 *g* for 1 h at 4°C. After centrifugation, the pellet was washed in 75% (v/v) ethanol and dissolved in 50 μL of RNase-free H_2_O. Next, the RNA was denatured at 75°C for 15 min and cooled for 2 min on ice. Before hybridizing the slot blots with the isolated RNA, the membrane was pre-hybridized with 20 mL of Church buffer (1 mM EDTA, 7% [w/v] SDS, 0.5 M NaHPO_4_ [pH 7.2]) in hybridization tubes at 65°C for 1 h. Hybridization was performed at 65°C overnight. Subsequently, the membrane was washed once with 1 × SSC and 0.2% (w/v) SDS for 10 min, and once with 0.5 × SSC and 0.2% (w/v) SDS for 10 min. After washing, the membrane was wrapped in a transparent foil and exposed to a storage phosphor screen for 5 days. The signals were detected using an Amersham Typhoon IP scanner.

### 5'-RACE experiments to map transcription start sites

TAP transcript 5'-end mapping in *Oenothera* chloroplasts was performed as previously described (Kühn et al., 2005). In brief, primary transcripts of bacteria and cell organelles have triphosphates at their 5′-ends, while processed transcripts possess monophosphates at the 5′-end. The tobacco acid pyrophosphatase (TAP) enzyme removes the additional phosphates from the 5'-end of primary transcripts. After this treatment, both primary and processed transcripts can serve as substrate for RNA ligase. This allows us to distinguish between primary and processed transcripts when +TAP and −TAP treated samples are compared. In −TAP samples, ligation products originating form primary transcripts are absent. Hence, to map the transcription start sites of the *psbB* operon and to distinguish them from processing sites in close proximity, a 5'-RACE from +TAP (Epicenter, Madison, WI, USA) and −TAP RNA samples was performed. For this, RNA samples of both treatments were ligated to an RNA linker (5'-GUGAUCCAACCGACGCGACAAGCUAAUGCAAGANNN-3'). After cDNA synthesis with a *psbB* gene-specific primer (psbB_cDNA_jn 5′-GCTGGCTGTCCATATAATGCATACAGC-3′), two PCRs were performed: the first PCR used the linker-specific primer RUMSH1 (5′-TGATCCAACCGACGCGAC-3′) and the *psbB*-specific primer psbB_cDNA_jn. The second PCR used the linker-specific nested primer RUMSH2 (5′-ACCGACGCGACAAGCTAATGC-3′) and the primer psbB_5prime_jn (5′-GGAAAGGGATTTTAGGCATACCAATCG-3′). PCR products (30 µL of PCR solution) were run on 1% agarose gels (w/v) and, prior to sequencing, cloned into pCR2.1-TOPO (Invitrogen).

### Ribosomal profiling

#### Monosome and polysome isolation

Polysome isolation from *Oenothera* leaves was performed based on a protocol modified from Ingolia et al. (2009) and Zoschke et al. (2013): 300 mg of leaf tissue were ground in liquid nitrogen and 3 mL of extraction buffer (40 mM Tris/acetate [pH 8.0], 0.2 M KCl, 10 mM MgCl_2_, 1% [v/v] Triton X-100, 2% [v/v] polyoxyethylene (10) tridecyl ether, 100 μg mL^−1^ chloramphenicol, 100 μg mL^−1^ cycloheximide, 0.2 M sucrose, 10 mM 2-mercaptoethanol, 15 mM boric acid, and 5 mM EGTA). The suspension was filtered through glass wool, centrifuged at 15,000 *g* for 10 min, and the supernatant was collected. For nuclease digestion, 300 U/mL RNaseI nuclease (Thermo Fisher Scientific) was added. The samples were incubated at room temperature for 1 h in a rotator. Subsequently, in the pre-purification step, the suspension was transferred onto a 2 mL sucrose cushion (40 mM Tris/acetate [pH 8.0], 100 mM KCl, 15 mM MgCl_2_, 100 µg mL^−1^ chloramphenicol, 100 µg mL^−1^ cycloheximide, 25% [w/v] sucrose, 5 mM 2-mercaptoethanol, 15 mM boric acid, and 5 mM EGTA). After ultracentrifugation for 1.5 h at 303,800 *g* at 4°C in a SW55 Ti rotor, the supernatant was discarded and the pellet, which contained the nuclease digested monosomes were stored at –80°C.

#### Ribosome footprint isolation

After nuclease digestion, the monosome pellet was suspended in 500 µL ribosome footprint isolation buffer (10 mM Tris [pH 8.0], 1 mM EDTA, [pH 8.0], 100 mM NaCl, 1% [w/v] SDS, 0.1 M EGTA [pH 8.0]) and transferred to a 2 mL centrifuge tube. To the monosome suspension 750 μL TRIzol™ was added, and the sample was vortexed and incubated for 10 min at room temperature. Subsequently, 150 μL of chloroform/isoamylalcohol was added; the sample was vortexed and centrifuged for 20 min at 20,000 *g* at room temperature. The supernatant was transferred into a 2 mL centrifuge tube containing 1 mL of isopropanol, and the RNA precipitated for 30 min at −20°C. Afterwards, the RNA was centrifuged for 40 min at 20,000 *g* at 4°C and the pellet was washed with 75% (v/v) ethanol. The pellet with the ribosome footprints was suspended in 20 μL of H_2_O. The RNA concentrations were measured using a NanoDrop 1000 spectrophotometer, and the RNA was stored at −80°C.

#### Denaturing RNA-PAGE for RNA purification

RNA was separated on denaturing 12% polyacrylamide gels (30% [v/v] Acryl/Bis™ [19:1], 1× TBE buffer [89 mM Tris [pH 8.0], 89 mM boric acid, and 2 mM EDTA], 80% [w/v] urea, 0.1% [w/v] ammonium persulfate, 0.6% [v/v] tetramethylethylenediamine). The RNA sample was dried out using a concentrator plus centrifuge (Eppendorf, Hamburg, Germany) and dissolved in 40 μL of loading buffer (90% [v/v] deionized formamide, 20 mM Tris [pH 7.5], 20 mM EDTA, 0.04% [w/v] xylene cyanol, 0.04% [w/v] bromphenol blue). After the incubation for 5 min at 45°C, the sample was denatured for 10 min at 75°C. For each lane, 30–40 µg of RNA dissolved in 40 µL loading buffer was loaded and 4 μL of Dynamarker Prestain Marker for small RNA Plus served as marker for the gel. The gel was electrophoresed in 1× TBE buffer at a constant power of 30 W per gel until the dye front reached the end of the gel. During the run, the gel apparatus was connected to a cold machine and cooled to 8°C (Pharmacia & Upjohn, Kalamazoo, MI, USA), which avoids gel melting and, especially for small nucleic acids, leads to better electrophoresis results by decreasing mobility rates. Then a region representing RNAs between 20 and 50 nt was excised and the RNA was eluted from the gel in 4 mL TESS solution per lane (10 mM Tris [pH 8.0], 1 mM EDTA [pH 8.0], 0.1 M NaCl, 0.2% [w/v] SDS) overnight in a rotator at 4°C. The solution was transferred to a new tube containing 4 mL phenol/chloroform/isoamyl alcohol (25:24:1) and 2.5 μL GlycoBlue™, vortexed and centrifuged for 5 min at 1,700 *g* at room temperature. Afterwards the supernatant was transferred into a new tube and RNA was precipitated at −20°C overnight by the addition of 2.5 volumes of 100% (v/v) ethanol. The next day, the sample was centrifuged at 15,000 *g* at 4°C for 1 h and the pellet was eluted in 500 μL H_2_O. Subsequently, the eluted ribosome footprints were transferred to a new tube containing 100 mM NaCl and 500 μL phenol/chloroform/isoamyl alcohol, vortexed and centrifuged at 15,300 *g* at room temperature for 20 min. Then the supernatant was transferred to a new tube containing chloroform/isoamyl alcohol, vortexed, and centrifuged at 15,300 *g* at room temperature for 20 min. To precipitate the RNA, the supernatant was transferred into new tubes with 2.5 volumes of 100% (v/v) ethanol and incubated overnight at –20°C. The next day, the sample was centrifuged at 20,000 *g* at 4°C for 1 h. Afterward the pellet was washed with 75% (v/v) ethanol, centrifuged at 20,000 *g* at 4°C for 10 min, and eluted in 20 μL of H_2_O. The size distributions of the ribosome footprints were measured using the Bioanalyzer (Agilent Technologies, Santa Clara, CA, USA) using the Agilent small RNA Kit, and the concentrations were determined with the Qubit 4 Fluorometer (Thermo Fisher Scientific) using the Qubit™ RNA Assay Kit. The ribosome footprints were stored at –80°C.

#### Validation

A detailed validation of the ribosomal profiling protocol established for evening primrose is reported in Kozul (2019) and will be published in a subsequent communication.

### NGS library preparation and sequencing

To prepare the ribosome footprints for the next-NGS library preparation, 5'-termini phosphorylation of the ribosome footprints was performed using the T4 PNK kit (polynucleotide kinase, Thermo Fisher Scientific), following the manufacturer’s instructions. Total RNA for RNA-seq libraries was isolated as described above. Subsequently, Ribo-seq and RNA-seq library preparations were performed according to the manufacturer’s protocols both for the LL and HL treatments in three replicates using the NEXTflex Small RNA-Seq Kit version 3 (PerkinElmer, Waltham, MA, USA) and Zymo Research (Freiburg, Germany) Zymo-Seq RiboFree Total RNA Library Kit, respectively. To provide details for the Ribo-seq library preparation, to 10.5 µL phosphorylated ribosome footprints 100 ng 3'-4N adenylated adapter and 5'-4N adenylated adapter were ligated, followed by the reverse transcription of the 3'- and 5'-adapter ligated RNA into a first strand synthesis product. The PCR products were subsequently amplified (18 cycles; 10 s at 98°C, 30 s at 60°C, and 15 s at 72°C) by using a universal primer and different barcoded primer for each sample. Afterward, a gel-free size selection and clean-up were performed. The size distribution of all final libraries was determined with the Bioanalyzer using the Agilent High Sensitivity DNA Kit, and the concentration was measured with the Qubit 4 Fluorometer using the Qubit™ dsDNA HS Assay (Thermo Fisher Scientific). Multiplexing and sequencing of the NGS libraries were performed at the Max Planck Institute for Molecular Genetics (Berlin, Germany) using an Illumina^®^ Nova Seq 6000 machine (SP flow cell for Ribo-seq and S1 flow cell for RNA-seq libraries, 100 bp single-end reads).

### NGS data analysis

Quality control of Ribo-seq (accessions GSM5288039 to GSM5288050) and RNA-seq (accessions GSM5288051 to GSM5288068) sequencing data, both included into the GEO Series GSE174154, was performed using FastQC version 0.11.8 (https://www.bioinformatics.babraham.ac.uk/projects/fastqc/). The 3′-adapter trimming was applied to both data sets using Flexbar version 2.5 (Dodt et al., 2012) with default parameters and the corresponding adapter–barcode-combined sequences. To remove a potential PCR bias from the Ribo-seq data set, the four-base 3'- and 5'-unique molecular identifiers (UMIs) were removed from the read sequences and attached to the read identifier in order to make them available for further usage, while sustaining correct alignment of the reads. All Ribo-seq data, as well as RNA-seq data for computing translation efficiency, were then aligned against plastome I (I-johSt = AJ271079.4) using STAR version 2.7.0a with slightly adapted indexing parameters (–genomeSAindexNbases 8 and –genomeChrBinNbits 17) (Dobin et al., 2013). Subsequently, samtools version 1.10 was used to remove secondary alignments, to sort and to index them, and to generate mapping, and gene-wise count statistics (flagstat and idxstat, respectively). Conversion of transcriptomic bam files (harboring only reads mapped to exons) to browser extensible data (bed) files was done using bamToBed from the bedtools version 2.29.2 (Quinlan and Hall, 2010), adding read length as additional column. Both file formats, bam and bed, contain coordinate-based information for reads mapped against a set of sequences. BAM is a compressed binary version of the Sequence Alignment/Map format and bed files contain less but the most important information for read alignments compared to the BAM format. See https://genome-euro.ucsc.edu/FAQ/FAQformat.html#format1 and https://github.com/samtools/hts-specs/blob/master/SAMv1.pdf, respectively, for format specifications and details. Usable reads for all follow-up analysis were defined by the following criteria: (1) removing tRNA and rRNA-associated reads as well as reads shorter than 20 bases and longer than 50 bases; (2) read de-duplication based on chromosomal location and same UMIs (by sorting bed files on the columns scaffold_id, start/end position and both columns having the UMIs using groupBy to take only the first entry of each group identical entries (-o first); (3) remain only reads with mapping quality equal to 255 (means unique mapped in the context of the STAR mapper). Resulting bed files were loaded into R to generate different types of plots.

Counts per million reads normalization was done on the total number of reads that mapped to protein-coding genes of the chloroplast. Gene length normalization to obtain reads per kb of exon per million reads values was applied by summing up the exon lengths for each gene in kilobase. Translational output and log_2_ ratio bar plots are based on gene-wise read counting in which only reads greater or equal to 25 and ≤35 were considered. Translational efficiency was computed by dividing the RF ratio by the RNA ratio where the ratios were calculated as follows: RF ratio (Ribo-seq sample1/sample2) and RNA ratio (RNA_seq sample1/sample2).

### Statistical analyses

All numerical results are reported as mean ± sd. Statistical significance of the difference between experimental groups was analyzed by unpaired *t* tests using GraphPad Prism software (Figure 2). Differences were considered statistically significant for *P* < 0.05 or *P* < 0.01. Two-way ANOVA with Tukey post-hoc testing was performed using SigmaPlot version 14.0 (Systat Software, San Jose, CA, USA; Table 1). Reports are provided in Supplemental File S4. RNA gel blots, immunoblots, and run-on analyses were repeated at least twice. Representative data are shown.

### Accession numbers

The following new GenBank Accession Numbers are reported in the manuscript. Novel chloroplast sequences are listed together with their metadata in Supplemental Table S1: KT881175.1, KX014625.1, MN807266.1, MN807267.1, MN812468.1, MN812469.1, MN812470.1, MN812471.1, MN812472.1, MN812473.1, MN812474.1, MN812475.1, MN812476.1, MN812477.1, MN812478.1, MN812479.1, MN812480.1, MN812481.1, MN812482.1, MN812483.1, MN812484.1, MN812485.1, MN812486.1, MN812487.1, MN812488.1, MN812489.1, MN812490.1, MN812491.1, MN812492.1, and MN812493.1. New Ribo-seq and RNA-seq Accession Numbers of the GEO Series GSE174154 are as follows: GSM5288039, GSM5288040, GSM5288041, GSM5288042, GSM5288043, GSM5288044, GSM5288045, GSM5288046, GSM5288047, GSM5288048, GSM5288049, GSM5288050, GSM5288051, GSM5288052, GSM5288053, GSM5288054, GSM5288055, GSM5288056, GSM5288057, GSM5288058, GSM5288059, GSM5288060, GSM5288061, GSM5288062, GSM5288063, GSM5288064, GSM5288065, GSM5288066, GSM5288067, and GSM5288068.

## Supplemental data

The following materials are available in the online version of this article.

**Supplemental Text.** Photosynthetic phenotype of AB-I plants, RNA gel blot analyses of *psbB* operon transcripts.

**Supplemental Figure S1.** Photosynthetic parameters of compatible AB-II and incompatible AB-I plants grown under LL or HL conditions

**Supplemental Figure S2.** RNA gel blot and RNA-seq analyses of the *clpP* and *psbB* operons in compatible (AA-I, AA-II, and AB-II) and incompatible material (AB-I).

**Supplemental Table S1.** Accession numbers, genome sizes, genetic information, and corresponding nuclear genotypes of *Oenothera* plastomes used for association mapping

**Supplemental Table S2.** Chloroplast mRNA editotype and cDNA mapping results of three *Oenothera* species from subsection *Oenothera*

**Supplemental Table S3.** Origin and collector information for the *Oenothera* strains used in this work

**Supplemental Table S4.** Chloroplast substitution lines, F1 hybrids, and corresponding wild-types used in this work

**Supplemental Table S5.** Oligonucleotides used for the generation of probes for RNA gel blot and run-on transcription analyses

**Supplemental File S1.** Multiple sequence alignment of the 46 *Oenothera* plastomes used for association mapping provided in the FASTA format (.txt file).

**Supplemental File S2.** Multiple sequence alignment and annotation of the 46 *Oenothera* plastomes used for association mapping provided as SeqMan Pro project file (DNASTAR) (.sqd file).

**Supplemental File S3.** Annotation of the alignment consensus of the 46 *Oenothera* plastomes used for association mapping provided in the GenBank format (.txt file).

**Supplemental File S4.** Two-way ANOVA reports generated using SigmaPlot14 software containing statistical analyses for Table 1, Figure 2B, and Supplemental Figure S2, A and C (.xls file).

## Supplementary Material

koab155_Supplementary_Data

## Notes

A.Z., D.K., M.A.S., J.N., F.G., A.F., I.M., and S.G. performed the experimental work. All authors analyzed and discussed data. S.G. designed the study and wrote the manuscript. R.B. participated in writing.

The author responsible for distribution of materials integral to the findings presented in this article in accordance with the policy described in the Instructions for Authors (https://academic.oup.com/plcell) is: Stephan Greiner (greiner@mpimp-golm.mpg.de).

## References

[koab155-B2] Arntz MA , Delph LF (2001) Pattern and process: evidence for the evolution of photosynthetic traits in natural populations. Oecologia 127: 455–467 28547482 10.1007/s004420100650

[koab155-B3] Barnard-Kubow KB , So N, Galloway LF (2016) Cytonuclear incompatibility contributes to the early stages of speciation. Evolution 70: 2752–2766 27677969 10.1111/evo.13075

[koab155-B4] Bogdanova VS (2020) Genetic and molecular genetic basis of nuclear-plastid incompatibilities. Plants 9: 23 10.3390/plants9010023 PMC7020172 31878042

[koab155-B5] Bogdanova VS , Zaytseva OO, Mglinets AV, Shatskaya NV, Kosterin OE, Vasiliev GV (2015) Nuclear-cytoplasmic conflict in pea (*Pisum sativum* L.) is associated with nuclear and plastidic candidate genes encoding acetyl-CoA carboxylase subunits. PLoS One 10: e0119835 25789472 10.1371/journal.pone.0119835 PMC4366379

[koab155-B6] Burrows PA , Sazanov LA, Svab Z, Maliga P, Nixon PJ (1998) Identification of a functional respiratory complex in chloroplasts through analysis of tobacco mutants containing disrupted plastid *ndh* genes. EMBO J 17: 868–876 9463365 10.1093/emboj/17.4.868 PMC1170436

[koab155-B7] Burton RS , Pereira RJ, Barreto FS (2013) Cytonuclear genomic interactions and hybrid breakdown. Ann Rev Ecol Evol Syst 44: 281–302

[koab155-B8] Chevalier F , Ghulam MM, Rondet D, Pfannschmidt T, Merendino L, Lerbs-Mache S (2015) Characterization of the *psbH* precursor RNAs reveals a precise endoribonuclease cleavage site in the *psbT*/*psbH* intergenic region that is dependent on *psbN* gene expression. Plant Mol Biol 88: 357–367 26012647 10.1007/s11103-015-0325-y

[koab155-B9] Chi W , He B, Mao J, Jiang J, Zhang L (2015) Plastid sigma factors: their individual functions and regulation in transcription. Biochim Biophys Acta 1847: 770–778 25596450 10.1016/j.bbabio.2015.01.001

[koab155-B10] Chiu WL , Sears BB (1985) Recombination between chloroplast DNAs does not occur in sexual crosses of *Oenothera*. Mol Gen Genet 198: 525–528 3859732 10.1007/BF00332951

[koab155-B11] Chiu WL , Stubbe W, Sears BB (1988) Plastid inheritance in *Oenothera*: organelle genome modifies the extent of biparental plastid transmission. Curr Genet 13: 181–189

[koab155-B12] Chou JY , Leu JY (2010) Speciation through cytonuclear incompatibility: insights from yeast and implications for higher eukaryotes. Bioessays 32: 401–411 20414898 10.1002/bies.200900162

[koab155-B13] Cleland RE (1935) Cyto-taxonomic studies on certain Oenotheras from California. Proc Am Philos Soc 75: 339–429

[koab155-B14] Cleland RE (1957) Chromosome structure in *Oenothera* and its effect on the evolution of the genus. Cytologia 22: 5–19

[koab155-B15] Cleland RE (1962) Plastid behaviour of the North American Euoenotheras. Planta 57: 699–712

[koab155-B16] Cleland RE (1972) *Oenothera* - Cytogenetics and Evolution. Academic Press Inc., London; New York, NY.

[koab155-B17] , Dietrich W, Wagner WL, Raven PH (1997) Systematics of *Oenothera* section *Oenothera* subsection *Oenothera* (Onagraceae). Syst Bot Monogr 50: 1–234

[koab155-B18] Dobin A , Davis CA, Schlesinger F, Drenkow J, Zaleski C, Jha S, Batut P, Chaisson M, Gingeras TR (2013) STAR: ultrafast universal RNA-seq aligner. Bioinformatics 29: 15–21 23104886 10.1093/bioinformatics/bts635 PMC3530905

[koab155-B19] Dodt M , Roehr JT, Ahmed R, Dieterich C (2012) FLEXBAR-flexible barcode and adapter processing for next-generation sequencing platforms. Biology 1: 895–905 24832523 10.3390/biology1030895 PMC4009805

[koab155-B20] Ekiz H , Kiral AS, Akçin A, Simsek L (1998) Cytoplasmic effects on quality traits of bread wheat (*Triticum aestivum* L.). Euphytica 100: 189–196

[koab155-B21] Fishman L , Willis JH (2006) A cytonuclear incompatibility causes anther sterility in *Mimulus* hybrids. Evolution 60: 1372–1381 16929654 10.1554/05-708.1

[koab155-B22] Fishman L , Sweigart AL (2018) When two rights make a wrong: the evolutionary genetics of plant hybrid incompatibilities. Annu Rev Plant Biol 69: 707–731 29505737 10.1146/annurev-arplant-042817-040113

[koab155-B23] Flood PJ (2019) Using natural variation to understand the evolutionary pressures on plant photosynthesis. Curr Opin Plant Biol 49: 68–73 31284076 10.1016/j.pbi.2019.06.001

[koab155-B24] Golczyk H , Massouh A, Greiner S (2014) Translocations of chromosome end-segments and facultative heterochromatin promote meiotic ring formation in evening primroses. Plant Cell 26: 1280–1293 24681616 10.1105/tpc.114.122655 PMC4001384

[koab155-B25] Greiner S , Bock R (2013) Tuning a ménage à trois: co-evolution and co-adaptation of nuclear and organellar genomes in plants. Bioessays 35: 354–365 23361615 10.1002/bies.201200137

[koab155-B26] Greiner S , Köhl K (2014) Growing evening primroses (*Oenothera*). Front Plant Sci 5: 38 24592268 10.3389/fpls.2014.00038 PMC3923160

[koab155-B27] Greiner S , Sobanski J, Bock R (2015) Why are most organelle genomes transmitted maternally? Bioessays 37: 80–94 25302405 10.1002/bies.201400110 PMC4305268

[koab155-B28] Greiner S , Rauwolf U, Meurer J, Herrmann RG (2011) The role of plastids in plant speciation. Mol Ecol 20: 671–691 21214654 10.1111/j.1365-294X.2010.04984.x

[koab155-B29] Greiner S , Wang X, Herrmann RG, Rauwolf U, Mayer K, Haberer G, Meurer J (2008a) The complete nucleotide sequences of the 5 genetically distinct plastid genomes of *Oenothera*, subsection *Oenothera*: II. A microevolutionary view using bioinformatics and formal genetic data. Mol Biol Evol 25: 2019–2030 18614526 10.1093/molbev/msn149 PMC2515874

[koab155-B30] Greiner S , Wang X, Rauwolf U, Silber MV, Mayer K, Meurer J, Haberer G, Herrmann RG (2008b) The complete nucleotide sequences of the five genetically distinct plastid genomes of *Oenothera*, subsection *Oenothera*: I. Sequence evaluation and plastome evolution. Nucleic Acids Res 36: 2366–2378 18299283 10.1093/nar/gkn081 PMC2367718

[koab155-B31] Grun P (1976) Cytoplasmic Genetics and Evolution. Columbia University Press, New York, NY.

[koab155-B32] Hager M , Biehler K, Illerhaus J, Ruf S, Bock R (1999) Targeted inactivation of the smallest plastid genome-encoded open reading frame reveals a novel and essential subunit of the cytochrome *b*_6_*f* complex. EMBO J. 18: 5834–5842 10545095 10.1093/emboj/18.21.5834 PMC1171649

[koab155-B33] Harrison JS , Burton RS (2006) Tracing hybrid incompatibilities to single amino acid substitutions. Mol Biol Evol 23: 559–564 16280539 10.1093/molbev/msj058

[koab155-B34] Harte C. (1994) *Oenothera* - Contributions of a Plant to Biology. Springer, Berlin, Heidelberg, Germany; New York, NY.

[koab155-B35] Hill GE , Havird JC, Sloan DB, Burton RS, Greening C, Dowling DK (2019) Assessing the fitness consequences of mitonuclear interactions in natural populations. Biol Rev 94: 1089–1104 30588726 10.1111/brv.12493 PMC6613652

[koab155-B36] Hoekstra LA , Julick CR, Mika KM, Montooth KL (2018) Energy demand and the context-dependent effects of genetic interactions underlying metabolism. Evol Lett 2: 102–113 30283668 10.1002/evl3.47 PMC6121862

[koab155-B37] Hollister JD , Greiner S, Johnson MTJ, Wright SI (2019) Hybridization and a loss of sex shape genome-wide diversity and the origin of species in the evening primroses (*Oenothera*, Onagraceae). New Phytol 224: 1372–1380 31309571 10.1111/nph.16053

[koab155-B38] Hollister JD , Greiner S, Wang W, Wang J, Zhang Y, Wong GKS, Wright SI, Johnson MTJ (2015) Recurrent loss of sex is associated with accumulation of deleterious mutations in *Oenothera*. Mol Biol Evol 32: 896–905 25534028 10.1093/molbev/msu345

[koab155-B39] Ingolia NT , Ghaemmaghami S, Newman JRS, Weissman JS (2009) Genome-wide analysis in vivo of translation with nucleotide resolution using ribosome profiling. Science 324: 218–223 19213877 10.1126/science.1168978 PMC2746483

[koab155-B40] Kahlau S , Aspinall S, Gray J, Bock R (2006) Sequence of the tomato chloroplast DNA and evolutionary comparison of Solanaceous plastid genomes. J Mol Evol 63: 194–207 16830097 10.1007/s00239-005-0254-5

[koab155-B41] Kirchhoff H , Mukherjee U, Galla HJ (2002) Molecular architecture of the thylakoid membrane: lipid diffusion space for plastoquinone. Biochemistry 41: 4872–4882 11939782 10.1021/bi011650y

[koab155-B42] Kozul D (2019) Systematic identification of loci determining chloroplast and nuclear genome incompatibilities in the evening primrose (*Oenothera*). PhD Thesis. Faculty of Mathematics and Natural Sciences University of Potsdam, Potsdam, Germany. p 140

[koab155-B43] Krech K , Fu HY, Thiele W, Ruf S, Schöttler MA, Bock R (2013) Reverse genetics in complex multigene operons by co-transformation of the plastid genome and its application to the open reading frame previously designated *psbN*. Plant J 75: 1062–1074 23738654 10.1111/tpj.12256

[koab155-B44] Kühn K , Weihe A, Börner T (2005) Multiple promoters are a common feature of mitochondrial genes in *Arabidopsis*. Nucleic Acids Res 33: 337–346 15653634 10.1093/nar/gki179 PMC546163

[koab155-B46] Lee HY , Chou JY, Cheong L, Chang NH, Yang SY, Leu JY (2008) Incompatibility of nuclear and mitochondrial genomes causes hybrid sterility between two yeast species. Cell 135: 1065–1073 19070577 10.1016/j.cell.2008.10.047

[koab155-B47] Leebens-Mack JH , Barker MS, Carpenter EJ, Deyholos MK, Gitzendanner MA, Graham SW, Grosse I, Li Z, Melkonian M, Mirarab S, et al (2019) One thousand plant transcriptomes and the phylogenomics of green plants. Nature 574: 679–685 31645766 10.1038/s41586-019-1693-2 PMC6872490

[koab155-B48] Lehwark P , Greiner S (2019) GB2sequin - a file converter preparing custom GenBank files for database submission. Genomics 111: 759–761 29842948 10.1016/j.ygeno.2018.05.003

[koab155-B49] Levin DA (2003) The cytoplasmic factor in plant speciation. Syst Bot 28: 5–11

[koab155-B50] Levy M , Levin DA (1975) Genic heterozygosity and variation in permanent translocation heterozygotes of the *Oenothera biennis* complex. Genetics 79: 493–512 17248680 10.1093/genetics/79.3.493 PMC1213289

[koab155-B51] Maddison WP , Maddison DR (2018) Mesquite: a modular system for evolutionary analysis. Version 3.61 http://www.mesquiteproject.org

[koab155-B52] Massouh A , Schubert J, Yaneva-Roder L, Ulbricht-Jones ES, Zupok A, Johnson MTJ, Wright SI, Pellizzer T, Sobanski J, Bock R, Greiner S (2016) Spontaneous chloroplast mutants mostly occur by replication slippage and show a biased pattern in the plastome of *Oenothera*. Plant Cell 28: 911–929 27053421 10.1105/tpc.15.00879 PMC4863383

[koab155-B53] Meiklejohn CD , Holmbeck MA, Siddiq MA, Abt DN, Rand DM, Montooth KL (2013) An incompatibility between a mitochondrial tRNA and its nuclear-encoded tRNA synthetase compromises development and fitness in *Drosophila*. PLoS Genet 9: e1003238 23382693 10.1371/journal.pgen.1003238 PMC3561102

[koab155-B54] Noordally ZB , Ishii K, Atkins KA, Wetherill SJ, Kusakina J, Walton EJ, Kato M, Azuma M, Tanaka K, Hanaoka M, et al (2013) Circadian control of chloroplast transcription by a nuclear-encoded timing signal. Science 339: 1316–1319 23493713 10.1126/science.1230397

[koab155-B55] Nováková E , Zablatzká L, Brus J, Nesrstová V, Hanáček P, Kalendar R, Cvrčková F, Majeský Ľ, Smýkal P (2019) Allelic diversity of acetyl coenzyme A carboxylase *accD*/*bccp* genes implicated in nuclear-cytoplasmic conflict in the wild and domesticated pea (*Pisum* sp.). Int J Mol Sci 20: 1773 30974846 10.3390/ijms20071773 PMC6480052

[koab155-B56] Ossenbühl F , Gohre V, Meurer J, Krieger-Liszkay A, Rochaix JD, Eichacker LA (2004) Efficient assembly of photosystem II in *Chlamydomonas reinhardtii* requires Alb3.1p, a homolog of *Arabidopsis* ALBINO3. Plant Cell 16: 1790–1800 15208384 10.1105/tpc.023226 PMC514161

[koab155-B252] Plöchinger M , Schwenkert S, von Sydow L, Schröder WP, Meurer J (2016) Functional update of the auxiliary proteins PsbW, PsbY, HCF136, PsbN, TerC and ALB3 in maintenance and assembly of PSII. Frontiers in Plant Science 7: 423–423 27092151 10.3389/fpls.2016.00423 PMC4823308

[koab155-B57] Porra RJ , Thompson WA, Kriedemann PE (1989) Determination of accurate extinction coefficients and simultaneous equations for assaying chlorophylls a and b extracted with four different solvents: verification of the concentration of chlorophyll standards by atomic absorption spectroscopy. Biochim Biophys Acta 975: 384–394

[koab155-B58] Postel Z , Touzet P (2020) Cytonuclear genetic incompatibilities in plant speciation. Plants 9: 487 32290056 10.3390/plants9040487 PMC7238192

[koab155-B59] Quinlan AR , Hall IM (2010) BEDTools: a flexible suite of utilities for comparing genomic features. Bioinformatics 26: 841–842 20110278 10.1093/bioinformatics/btq033 PMC2832824

[koab155-B60] Rand DM , Mossman JA (2020) Mitonuclear conflict and cooperation govern the integration of genotypes, phenotypes and environments. Philos Trans Royal Soc B Biol Sci 375: 20190188 10.1098/rstb.2019.0188 PMC6939372 31787039

[koab155-B62] Rauwolf U , Golczyk H, Meurer J, Herrmann RG, Greiner S (2008) Molecular marker systems for *Oenothera* genetics. Genetics 180: 1289–1306 18791241 10.1534/genetics.108.091249 PMC2581935

[koab155-B63] Raven PH , Dietrich W, Stubbe W (1979) An outline of the systematics of *Oenothera* subsect *Euoenothera* (Onagraceae). Syst Bot 4: 242–252

[koab155-B64] Rostański K (1982) The species of *Oenothera* L. in Britain. Watsonia 14: 1–34

[koab155-B65] Rostański K (1985) Zur Gliederung der Subsektion *Oenothera* (Sektion *Oenothera*, *Oenothera* L., Onagraceae). Feddes Repert 96: 3–14

[koab155-B66] Rostański K , Rostański A, Gerold-Śmietańska I, Wasowicz P (2010) Evening-Primroses (*Oenothera*) Occurring in Europe. Polish Academy of Science, Władysław Szafer Institute of Botany, Krakow, Poland.

[koab155-B67] Rott M , Martins NF, Thiele W, Lein W, Bock R, Kramer DM, Schöttler MA (2011) ATP synthase repression in tobacco restricts photosynthetic electron transport, CO_2_ assimilation, and plant growth by overacidification of the thylakoid lumen. Plant Cell 23: 304–321 21278125 10.1105/tpc.110.079111 PMC3051256

[koab155-B68] Sambatti JBM , Ortiz-Barrientos D, Baack EJ, Rieseberg LH (2008) Ecological selection maintains cytonuclear incompatibilities in hybridizing sunflowers. Ecol Lett 11: 1082–1091 18643842 10.1111/j.1461-0248.2008.01224.x PMC2737365

[koab155-B69] Schmitz-Linneweber C , Kushnir S, Babiychuk E, Poltnigg P, Herrmann RG, Maier RM (2005) Pigment deficiency in nightshade/tobacco cybrids is caused by the failure to edit the plastid ATPase alpha-subunit mRNA. Plant Cell 17: 1815–1828 15894714 10.1105/tpc.105.032474 PMC1143079

[koab155-B70] Schöttler MA , Tóth SZ (2014) Photosynthetic complex stoichiometry dynamics in higher plants: environmental acclimation and photosynthetic flux control. Front Plant Sci 5: 188 24860580 10.3389/fpls.2014.00188 PMC4026699

[koab155-B71] Schöttler MA , Kirchhoff H, Weis E (2004) The role of plastocyanin in the adjustment of the photosynthetic electron transport to the carbon metabolism in tobacco. Plant Physiol 136: 4265 15563617 10.1104/pp.104.052324 PMC535856

[koab155-B72] Schöttler MA , Flügel C, Thiele W, Bock R (2007a) The plastome-encoded PsaJ subunit is required for efficient photosystem I excitation, but not for plastocyanin oxidation in tobacco. Biochem J 403: 251–260 17209805 10.1042/BJ20061573 PMC1874242

[koab155-B73] Schöttler MA , Flügel C, Thiele W, Bock R (2007b) Knock-out of the plastid-encoded PetL subunit results in reduced stability and accelerated leaf age-dependent loss of the cytochrome *b*_6_*f* complex. J Biol Chem 282: 976–985 17114182 10.1074/jbc.M606436200

[koab155-B74] Schötz F (1954) Über Plastidenkonkurrenz bei *Oenothera*. Planta 43: 182–240

[koab155-B75] Schumacher E , Steiner EE (1993) Cytological analysis of complex-heterozygotes in populations of *Oenothera grandiflora* (Onagraceae) in Alabama. Plant Syst Evol 184: 77–87

[koab155-B76] Schumacher E , Steiner EE, Stubbe W (1992) The complex-heterozygotes of *Oenothera grandiflora* L'Her. Bot Acta 105: 375–381

[koab155-B77] Schwenkert S , Legen J, Takami T, Shikanai T, Herrmann RG, Meurer J (2007) Role of the low-molecular-weight subunits PetL, PetG, and PetN in assembly, stability, and dimerization of the cytochrome *b*_6_*f* complex in tobacco. Plant Physiol 144: 1924–1935 17556510 10.1104/pp.107.100131 PMC1949900

[koab155-B78] Schwenkert S , Umate P, Dal Bosco C, Volz S, Mlcochova L, Zoryan M, Eichacker LA, Ohad I, Herrmann RG, Meurer J (2006) PsbI affects the stability, function, and phosphorylation patterns of photosystem II assemblies in tobacco. J Biol Chem 281: 34227–34238 16920705 10.1074/jbc.M604888200

[koab155-B79] Shimizu M , Kato H, Ogawa T, Kurachi A, Nakagawa Y, Kobayashi H (2010) Sigma factor phosphorylation in the photosynthetic control of photosystem stoichiometry. Proc Natl Acad Sci U S A 107: 10760–10764 20498041 10.1073/pnas.0911692107 PMC2890857

[koab155-B80] Simon M , Durand S, Pluta N, Gobron N, Botran L, Ricou A, Camilleri C, Budar F (2016) Genomic conflicts that cause pollen mortality and raise reproductive barriers in *Arabidopsis thaliana*. Genetics 203: 1353–1367 27182945 10.1534/genetics.115.183707 PMC4937478

[koab155-B81] Sloan DB , Havird JC, Sharbrough J (2017) The on-again, off-again relationship between mitochondrial genomes and species boundaries. Mol Ecol 26: 2212–2236 27997046 10.1111/mec.13959 PMC6534505

[koab155-B82] Sobanski J , Giavalisco P, Fischer A, Kreiner JM, Walther D, Schöttler MA, Pellizzer T, Golczyk H, Obata T, Bock R, et al (2019) Chloroplast competition is controlled by lipid biosynthesis in evening primroses. Proc Natl Acad Sci U S A 116: 5665–5674 30833407 10.1073/pnas.1811661116 PMC6431223

[koab155-B83] Steiner EE , Stubbe W (1984) A contribution to the population biology of *Oenothera grandiflora* L’Her. Am J Bot 71: 1293–1301

[koab155-B84] Steiner EE , Stubbe W (1986) *Oenothera grandiflora* revisited: a new view of its population structure. Bull Torrey Bot Club 113: 406–412

[koab155-B85] Stubbe W (1959) Genetische Analyse des Zusammenwirkens von Genom und Plastom bei *Oenothera*. Zeitschr Vererbung 90: 288–298

[koab155-B86] Stubbe W (1960) Untersuchungen zur genetischen analyse des plastoms von *Oenothera*. Zeitschr Bot 48: 191–218

[koab155-B87] Stubbe W (1963) Die Rolle des Plastoms in der Evolution der Oenotheren. Ber Dtsch Bot Ges 76: 154–167

[koab155-B88] Stubbe W (1964) The role of the plastome in evolution of the genus *Oenothera*. Genetica 35: 28–33

[koab155-B89] Stubbe W (1989) *Oenothera* - An ideal system for studying the interaction of genome and plastome. Plant Mol Biol Rep 7: 245–257

[koab155-B90] Stubbe W , Raven PH (1979a) Genetic self-incompatibility in *Oenothera* subsect *Euoenothera*. Science 204: 327 17800360 10.1126/science.204.4390.327

[koab155-B91] Stubbe W , Raven PH (1979b) A genetic contribution to the taxonomy of *Oenothera* sect. *Oenothera* (including subsection *Euoenothera*, *Emersonia*, *Raimannia* and *Munzia*). Plant Syst Evol 133: 39–59

[koab155-B92] Stubbe W , Steiner E (1999) Inactivation of pollen and other effects of genome-plastome incompatibility in *Oenothera*. Plant Syst Evol 217: 259–277

[koab155-B93] Takenaka M , Zehrmann A, Verbitskiy D, Härtel B, Brennicke A (2013) RNA editing in plants and its evolution. Ann Rev Genet 47: 335–352 24274753 10.1146/annurev-genet-111212-133519

[koab155-B251] Thompson JD , Higgins DG, Gibson TJ (1994) CLUSTAL W: improving the sensitivity of progressive multiple sequence alignment through sequence weighting, position-specific gap penalties and weight matrix choice. Nucleic Acids Research 22: 4673–4680 7984417 10.1093/nar/22.22.4673 PMC308517

[koab155-B94] Tillich M , Lehwark P, Ulbricht-Jones ES, Fischer A, Pellizzer T, Bock R, Greiner S (2017) GeSeq - versatile and accurate annotation of organelle genomes. Nucleic Acids Res 45: W6–W11 28486635 10.1093/nar/gkx391 PMC5570176

[koab155-B95] Torabi S , Umate P, Manavski N, Plöchinger M, Kleinknecht L, Bogireddi H, Herrmann RG, Wanner G, Schröder WP, Meurer J (2014) PsbN is required for assembly of the photosystem II reaction center in *Nicotiana tabacum*. Plant Cell 26: 1183–1199 24619613 10.1105/tpc.113.120444 PMC4001377

[koab155-B96] Umate P , Schwenkert S, Karbat I, Bosco CD, Mlcòchová L, Volz S, Zer H, Herrmann RG, Ohad I, Meurer J (2007) Deletion of *psbM* in tobacco alters the QB site properties and the electron flow within photosystem II. J Biol Chem 282: 9758–9767 17261590 10.1074/jbc.M608117200

[koab155-B97] Wasmund O (1980) Cytogenetische Untersuchung zur Systematik einiger Sippen der Subsektion *Euoenothera* der Gattung *Oenothera* (*Onagraceae*). State Examination Thesis. Institute of Botany Heinrich Heine University Düsseldorf, Düsseldorf, Germany, p 103.

[koab155-B98] Wasmund O (1990) Cytogenetic investigation on *Oenothera nutans* (Onagraceae). Plant Syst Evol 169: 69–80

[koab155-B99] Wasmund O , Stubbe W (1986) Cytogenetic investigations on *Oenothera wolfii* (Onagraceae). Plant Syst Evol 154: 79–88

[koab155-B100] Woźniak-Chodacka M (2018) A revision of taxonomic relation between *Oenothera perangusta* and *O. ersteinensis* (Onagraceae) based on morphometric research and statistical analyses. Phytotaxa 383: 55–74

[koab155-B101] Zghidi-Abouzid O , Merendino L, Buhr F, Malik Ghulam M, Lerbs-Mache S (2011) Characterization of plastid *psbT* sense and antisense RNAs. Nucleic Acids Res 39: 5379–5387 21421558 10.1093/nar/gkr143 PMC3141253

[koab155-B102] Zhang J , Ruhlman TA, Sabir J, Blazier JC, Jansen RK (2015) Coordinated rates of evolution between interacting plastid and nuclear genes in Geraniaceae. Plant Cell 27: 563–573 25724640 10.1105/tpc.114.134353 PMC4558654

[koab155-B103] Zoschke R , Watkins KP, Barkan A (2013) A rapid ribosome profiling method elucidates chloroplast ribosome behavior *in **vivo*. Plant Cell 25: 2265–2275 23735295 10.1105/tpc.113.111567 PMC3723625

[koab155-B104] Zupok A (2015) The *psbB* operon is a major locus for plastome-genome incompatibility in *Oenothera*. PhD Thesis. Faculty of Mathematics and Natural Sciences University of Potsdam, Potsdam, Germany, p 108.

