# Supplementary material for: A photosynthesis operon in the chloroplast genome drives speciation in evening primroses
Source: Plant Cell. 2021 May 28;33(8):2583–601. doi: 10.1093/plcell/koab155 (PMC8408503; doi:10.1093/plcell/koab155)
Supplement: koab155_Supplementary_Data [file koab155_supplementary_data.zip › tpc.00618.2020-s02.pdf]

SUPPLEMENTAL TEXT

Photosynthetic phenotype of AB-I plants

To understand the yellow-green (*lutescent*) AB-I phenotype (Figure 2A), we performed a detailed characterization of its photosynthetic parameters. It appeared that the photosynthetic apparatus of *Oenothera* AB-I plants suffers from light-dependent damage. When plants were grown under three different light intensities (300, 450 and 600 µE m^{-2} s^{-1}), no damage to the photosynthetic apparatus of AB-I occurred at 300 µE m^{-2} s^{-1} (see below). At 600 µE m^{-2} s^{-1}, however, destruction of the photosynthetic apparatus was already so massive that it precluded a detailed photosynthetic characterization.

We, therefore, characterized compatible AB-II and incompatible AB-I plants grown at 300 and 450 µE m^{-2} s^{-1}, designated as low light (LL) and high light (HL), respectively, where pronounced differences were observed. Under LL, function and composition of the photosynthetic apparatus were indistinguishable between AB-II and AB-I. As judged from photosynthetic complex quantification by difference absorbance measurements normalized to a leaf area basis, the contents of photosystem II (PSII), cytochrome b_6f complex (Cytb_6f), the mobile redox carrier plastocyanin (PC) and photosystem I (PSI) were indistinguishable between the genotypes (Figure 2B). Also, the maximum quantum efficiency of PSII in the dark-adapted state (F_V/F_M) was identical (0.79), clearly showing that no photoinhibition of PSII occurred under these conditions. The same was true for the total chlorophyll content per leaf area (Supplemental Figure 1A).

By contrast, the increase in light intensity to HL resulted in drastic changes in photosynthetic complex accumulation, F_V/F_M and chlorophyll content in the two genotypes. AB-II plants responded to the increased growth light intensity by strongly increasing their chlorophyll content and the contents of all redox-active components of the electron transport chain, ranging from a more than two-fold increase of PSII content to a 50% increase in PSI content (Figure 2B). These increases represent the typical light-acclimation response that occurs when plants previously grown under light-limited conditions are transferred to a higher light intensities (Schöttler and Tóth, 2014; [1]). AB-I plants were incapable of performing this light acclimation response efficiently. Their PSII content increased only by 40%, and the strong decrease in F_V/F_M suggested that a substantial number of PSII centers were photodamaged. Also, contents of Cytb_6f complex and PC increased to a much lesser

degree than in AB-II, and PSI content remained essentially unaltered (Figure 2B). Consistent with these observations, chlorophyll content per leaf area increased only by 25% in AB-I (Supplemental Figure 1A).

To assess possible consequences of these different light acclimation responses of AB-II and AB-I on the relative antenna cross sections of the two photosystems, chlorophyll-*a* fluorescence emission spectra at 77K were recorded (Supplemental Figure 1B). For better comparability, the spectra were normalized to the PSII emission maximum at 687 nm wavelength. Under LL, AB-II had a higher PSI emission signal than AB-I, consistent with its slightly higher ratio of PSI to PSII. With increasing light intensity, the photosystem I/light harvesting complex I (PSI-LHCI) the emission signal, peaking at 733 nm wavelength, decreased in both AB-II and AB-I, consistent with the more pronounced increase in PSII content in both genotypes. No indications for the presence of free, uncoupled light-harvesting complex I (LHCI) or light-harvesting complex II (LHCII) were observed. These would be expected to result in additional emission signals at 680 nm wavelength (indicative of free LHCII), or between 705 and 730 nm wavelength (indicative of the presence of uncoupled LHCI) (Krause and Weis, 1991; Albus et al., 2010; Krech et al. 2012; [2-4]). Therefore, the decreased F_V/F_M ratio of AB-I under HL (Figure 2B) cannot be explained by the presence of uncoupled antenna, but has to be attributed to photoinhibition of the PSII reaction centers themselves.

Finally, chloroplast ATP synthase (ATPase) activity and accumulation were assessed (Supplemental Figures 1C and 1D). Dark-interval relaxation kinetics of the electrochromic shift, a measure for the proton motive force across the thylakoid membrane, were used to determine the thylakoid conductivity for protons (gH^+). The latter, in turn, is a proxy for ATPase activity (Rott et al. 2011; Cruz et al. 2001; [5,6]). Interestingly, as judged from immunoblot analyses of the AtpA protein, a core subunit of the ATP synthase complex, a slight reduction of ATP synthase content might be present in AB-I plants, at least under low light conditions (Supplemental Figure 1D). This, however, did not lead to significant differences in ATP synthase activity between the genotypes (Supplemental Figure 1C). Moreover, we did not observe significant differences between the growth light intensities. In this context, it should be mentioned that, in contrast to the complex quantifications expressed on a leaf area basis, gH^+ is a measure for ATP synthase activity per thylakoid membrane and not per leaf area. Hence, since chlorophyll content increased with light intensity, it is likely that also total ATP synthase activity per leaf

area was higher in plants grown under HL compared to plants grown under LL. Nonetheless, this effect is independent of the compatible/incompatible situation of AB-II/ AB-I plants.

In summary, AB-I plants display a light-dependent photosynthesis phenotype that cannot be attributed to a single component of the electron transport chain. However, the phenotype is independent of ATP synthase and PC function.

RNA gel blot analyses of *psbB* operon transcripts

Transcript analysis of the *psbB* operon showed a clear decrease in mRNA accumulation in AB-I plants under HL conditions, whereas no differences were detected for AB-I plants in LL (Supplemental Figures 2A and 2C). Upon hybridization with a *psbB* probe, four major *psbB*-containing transcript species were detected. The pentacistronic *psbB-psbT-psbH-petB-petD* transcript (5.6 kb with the introns of *petB* and *petD*, and 3.9 kb without these introns), the tricistronic *psbB-psbT-psbH* transcript (2.6 kb) and the dicistronic mature *psbB-psbT* transcript (1.8 kb). For details on the maturation of the *psbB* operon, see Westhoff and Herrman (1988) [7]. Surprisingly, a processing defect was detected in AB-I plants under HL conditions in that the AB-I plants lack the 3.9 kb transcript with the *petB* and *petD* introns spliced out (Supplemental Figure 2C, boxed in yellow). The same transcript species were detected when blots were hybridized to a *psbT* probe. Hybridization to a *psbH* probe revealed a down-regulation of all *psbH* transcripts. Similarly to hybridization with the *psbB* probe, virtual absence of the 3.9 kb transcripts was observed. For both *petB* and *petD*, a decrease of polycistronic and monocistronic transcripts was observed. Both *petB* and *petD* mRNAs appear to accumulate to higher levels in AB-I at LL. Again, absence of the 3.9 kb transcript species was confirmed.

In summary, the data show that expression of the entire *psbB* operon is affected by the deletion. In the absence of any polymorphism between plastome I and II in the whole operon (Supplemental Files 1-3), the observed processing defect is likely to be a secondary consequence of the deletion. The precise molecular mechanism underlying this effect is currently unknown.

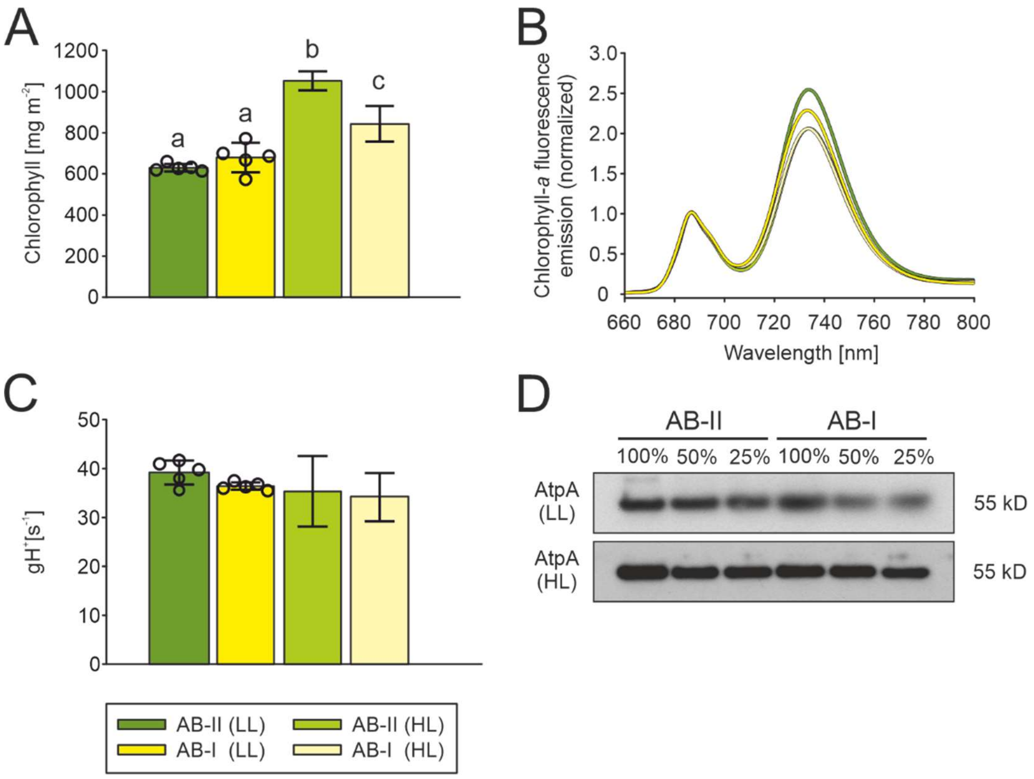

Supplemental Figure 1. Photosynthetic parameters of compatible AB-II and incompatible AB-I plants grown under LL or HL conditions (Supports Figure 2).

(A) Chlorophyll content per leaf area. 6-8 plants grown alongside were analyzed. For AB-II (LL) and AB-I (LL) data points are shown. Bars represent mean values ±SD. Different lowercase letters indicate significant difference ($P < 0.05$) according to two-way ANOVA with interactions followed by Tukey post-hoc testing (Supplemental File 4).

(B) Chlorophyll-*a* fluorescence emission spectra at 77K (n = 4-6 plants grown alongside).

(C) Thylakoid membrane conductivity for protons (gH^+) as proxy for ATP synthase activity. 6-8 plants grown alongside were analyzed. For AB-II (LL) and AB-I (LL) data points are shown. Bars represent mean values ±SD. For AB-II (LL) and AB-I (LL) data points are shown.

(D) Immunoblot analysis of the AtpA protein, a core subunit of the ATP synthase complex. Samples are normalized to chlorophyll content. This experiment was performed three times independently with similar results.

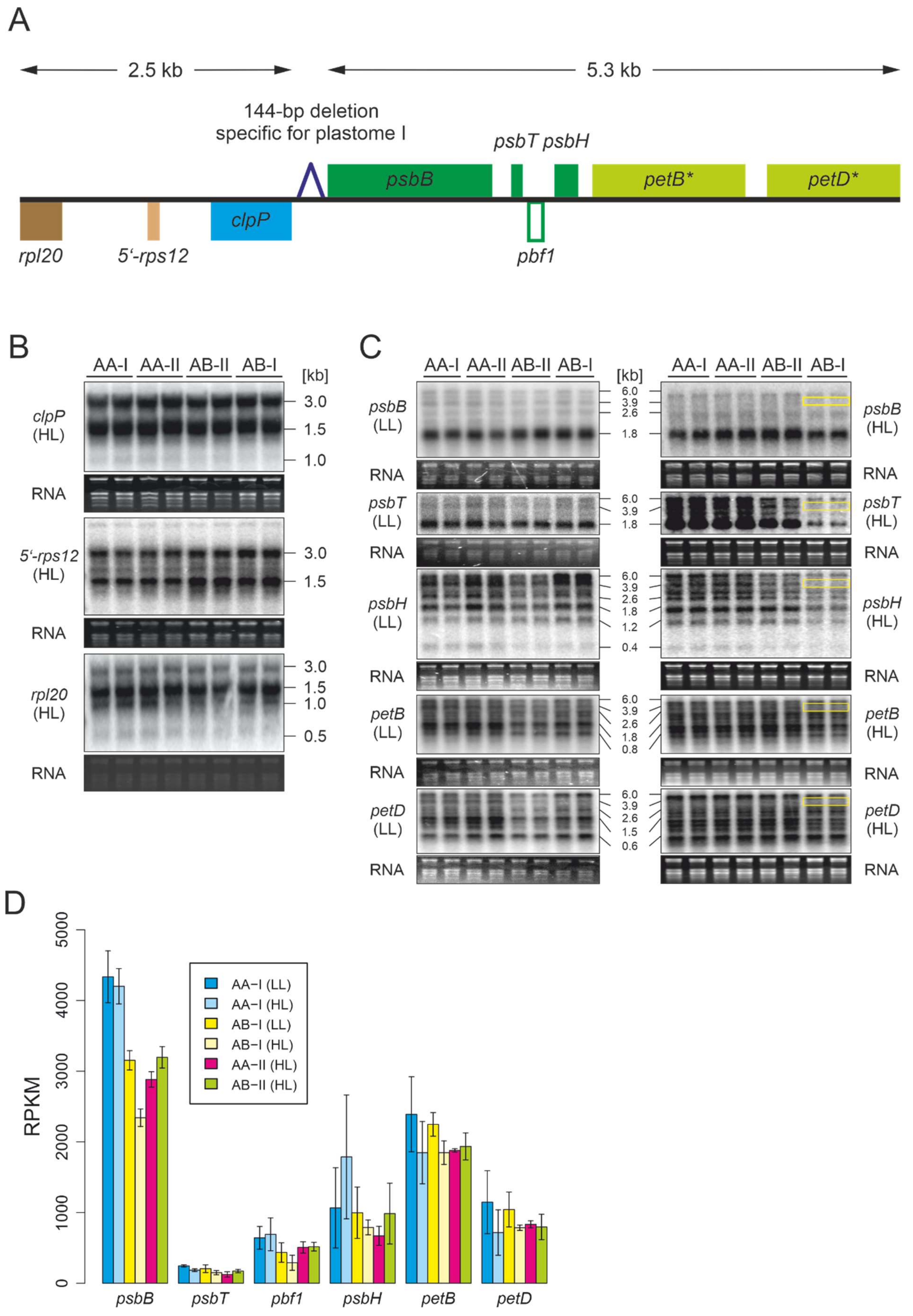
A
2.5 kb
5.3 kb
144-bp deletion
specific for plastome I
psbT psbH
psbB
petB*
petD*
rpl20
5'-rps12
clpP
pbf1
B
AA-I AA-II AB-II AB-I
[kb]
clpP (HL)
3.0
1.5
1.0
RNA
5'-rps12 (HL)
3.0
1.5
RNA
rpl20 (HL)
3.0
1.5
1.0
0.5
RNA
C
AA-I AA-II AB-II AB-I
[kb]
AA-I AA-II AB-II AB-I
psbB (LL)
6.0
3.9
2.6
1.8
psbB (HL)
RNA
psbT (LL)
6.0
3.9
1.8
psbT (HL)
RNA
psbH (LL)
6.0
3.9
2.6
1.8
1.2
0.4
psbH (HL)
RNA
petB (LL)
6.0
3.9
2.6
1.8
0.8
petB (HL)
RNA
petD (LL)
6.0
3.9
2.6
1.5
0.6
petD (HL)
RNA
D
RPKM
0
1000
2000
3000
4000
5000
AA−I (LL)
AA−I (HL)
AB−I (LL)
AB−I (HL)
AA−II (HL)
AB−II (HL)
psbB
psbT
pbf1
psbH
petB
petD

Supplemental Figure 2. RNA gel blot and RNA-seq analyses of the *clpP* and *psbB* operons in compatible (AA-I, AA-II, AB-II) and incompatible (AB-I) material (Supports Figure 4).

(A) Sequence context and position of the deletion specific to plastome I. Note that *clpP* is co-transcribed with exon 1 of the trans-spliced gene *rps12* (*5'-rps12*) and with *rpl20*. The intron-containing genes *petB* and *petD* are marked by asterisks.

(B) RNA gel blot analyses of *clpP* operon transcripts in plants grown under HL conditions. Northern blots were performed two times independently with similar results.

(C) RNA gel blot analyses of *psbB* operon transcripts under LL and HL conditions. The processing defect is boxed in yellow. This experiment was performed two times independently with similar results.

(D) RNA-seq analyses of *psbB* operon transcripts, including *pbf1* encoded on the opposite strand, under LL and HL conditions. Please note that the RPKM values largely confirm the RNA gel blot analysis. The experiment is based on three replicates from a pool of about 20 individuals per genotype. Bars represent mean values ±SD.

Supplemental Table 1. Accession numbers, genome sizes, genetic information, and corresponding nuclear genotype of *Oenothera* plastomes used for association mapping.

Species	Strain[1)]	Plastome	Plastome type	Nuclear genome type	Reference genotype	Size [bp]	GenBank/EMBL accession number	Reference plastome
O. elata ssp. *elata*	elata Cholula	I-eCho	I	AA	[8-10]	165,373	MN807266.1	this work
O. elata ssp. *elata*	elata Guatemala	I-eGua	I	AA	[8-10]	165,520	MN812469.1	this work
O. elata ssp. *elata*	elata Puebla	I-ePue	I	AA	[8-10]	165,551	MN812470.1	this work
O. elata ssp. *elata*	elata Toluca	I-eTol	I	AA	[8-10]	165,403	KT881169.2	[11]
O. elata ssp. *hookeri*	franciscana de Vries	I-frandV	I	AA	[9,12-14]	165,749	MN812471.1	this work
O. elata ssp. *hookeri*	hookeri de Vries	I-hookdV	I	AA	[9,12-14]	165,359	KT881170.1	[11,15]
O. elata ssp. *hookeri*	johansen Standard	I-johSt	I	AA	[9,14,16]	165,899	AJ271079.4	[11,15,17-19]
O. villosa ssp. *strigosa*	strigosa de Vries	I-strdV	I	AA	[8,9,14]	165,138	MN812484.1	this work
O. villosa ssp. *villosa*	bauri Standard	I-bauriSt	I	AA	[9,12,13]	164,312	KX687910.1	[15]
O. villosa ssp. *villosa*	cockerelli de Vries	I-cockdV	I	AA	[8,9,14]	165,666	MN812468.1	this work
O. villosa ssp. *villosa*	mollis Standard	I-molSt	I	AA	[8]	165,760	MN812483.1	this work
O. villosa ssp. *villosa*	strigosa Iowa 2	I-strIo2	I	AA	[8,9,14]	165,451	MN807267.1	this work
O. villosa ssp. *villosa*	strigosa Iowa 6	I-strIo6	I	AA	[8,14]	165,619	MN812485.1	this work
O. villosa ssp. *villosa*	villosa Madeleine	I-vilMad	I	AA	[20]	165,726	MN812488.1	this work
O. wolfii	wolfii Crescent City 1	I-wolCC1	I	AA	[20,21]	165,589	MN812490.1	this work
O. wolfii	wolfii Crescent City 3	I-wolCC3	I	AA	[20,21]	165,590	MN812491.1	this work
O. wolfii	wolfii Lufftenholtz	I-wolLu	I	AA	[20,21]	165,021	MN812492.1	this work
O. wolfii	wolfii Petrolia	I-wolPe	I	AA	[20,21]	165,153	MN812493.1	this work
O. biennis x glazioviana	conferta Standard	II-conSt	II	AB	[8]	164,723	MN812473.1	this work
O. biennis x glazioviana	coronifera Standard	II-corSt	II	AB	[8]	164,733	MN812474.1	this work
O. biennis x O. villosa	hoelscheri Standard	II-hoeSt	II	AB	[8]	164,796	MN812475.1	this work
O. biennis	biennis Muenchen	II-biM	II	AB	[12,13]	164,797	KU521375.1	[15]
O. biennis	biennis Shuswap Lake	II-biSL	II	AB	[20]	164,822	MN812472.1	this work
O. biennis	purpurata Standard	II-purSt	II	AA	[9,12,13]	164,832	MN812482.1	this work

Supplemental Table 1. (continued)

Species	Strain[1)]	Plastome	Plastome type	Nuclear genome type	Reference genotype	Size [bp]	GenBank/EMBL accession number	Reference plastome
O. biennis	nuda Standard	II-nudaSt	II	AB	[8]	165,112	MN812481.1	this work
O. biennis	rubricaulis Thorn	II-rcauTh	II	AB	[8]	164,768	KX687914.1	[15]
O. biennis	suaveolens Standard	II-suavSt	II	AB	[12,13]	164,796	KX687915.1	[15]
O. biennis	suaveolens Grado	II-suavG	II	AB	[12,13]	164,796	EU262889.2	[11,15,18,19]
O. biennis	suaveolens *xanthodermis* Fuenfkirchen	II-suavFue	II	AB	[12,13]	164,796	KT881175.1	[11]
O. biennis	suaveolens *sulfurea* Friedrichshagen	II-suavFr	II	AB	[12,13]	164,784	MH168560.1	this work
O. biennis	chicaginensis Colmar	III-chicCol	III	BA	[8,9]	166,074	MN812480.1	this work
O. biennis	chicaginensis de Vries	III-chicdV	III	BA	[12-14]	166,336	KX687913.1	[15]
O. biennis	biennis-1 Citronelle	III-bi1Cit	III	BA	[9,12-14]	165,845	MN812476.1	this work
O. biennis	biennis-1 Hot Springs	III-bi1HS	III	BA	[9,12-14]	166,087	MN812477.1	this work
O. biennis	biennis-1 Paducah	III-bi1Pad	III	BA	[9,12-14]	166,159	MN812478.1	this work
O. biennis	biennis-1 Walkerton	III-bi1Wal	III	BA	[9,12-14]	166,198	MN812479.1	this work
O. glazioviana	*r*/*r*-lamarckiana Sweden	III-lamS	III	AB	[12,13]	165,359	EU262890.2	[11,15,18,19]
O. grandiflora	grandiflora Tuscaloosa	III-graTus	III	BB	[16,22]	166,697	KX014625.1	this work
O. oakesiana	ammophila Standard	IV-ammSt	IV	AC	[12,13]	163,575	KT881176.1	[11,15]
O. oakesiana	ammophila Sylt	IV-ammSy	IV	AC	[8]	163,691	MN812486.1	this work
O. oakesiana	germanica Standard	IV-gerSt	IV	AC	[8]	163,507	MN812487.1	this work
O. oakesiana	parviflora-1 Iron Mountain	IV-par1IM	IV	AC	[20]	163,507	MN812489.1	this work
O. oakesiana	*r*/*r*-syrticola Ulm	IV-syrtU	IV	AC	[12,13]	163,578	KX687918.1	[15]
O. parviflora	atrovirens Standard	IV-atroSt	IV	BC	[12-14]	163,367	EU262891.2	[11,15,18,19]
O. parviflora	silesiaca Standard	IV-silSt	IV	BC	[12,13]	163,398	KX687917.1	[15]
O. parviflora	rubricuspis Standard	IV-rcuSt	IV	BC	[8]	163,396	KX687916.1	[15]

[1)] For details on the corresponding *Oenothera* strains, see Supplemental Table 3.

Supplemental Table 2. Chloroplast mRNA editotype and cDNA mapping results of three *Oenothera* species from subsection *Oenothera*. For details, see Methods. See Supplemental References [23,24] for additional data[1)].

Gene	Codon Position	Codon	AA Exchange	Editing %	Plastome	Reference	Ref. Position[2)]	Ref. Base	Called Base	Depth	A Count	C Count	G Count	T Count
atpA	264	CCC>CtC	P>L	92.96%	I-johSt	AJ271079.4	55408	C	T	256	0	-	0	238
				97.82%	II-suavG	EU262889.2	55256	C	T	46	0	-	0	45
				100.00%	III-graTus	KX014625.1	55291	C	T	109	0	-	0	109
atpF	31	CCA>CtA	P>L	86.86%	I-johSt	AJ271079.4	53343	C	T	335	0	-	0	290
				98.79%	II-suavG	EU262889.2	53194	C	T	83	0	-	0	82
				96.99%	III-graTus	KX014625.1	53230	C	T	632	0	-	0	613
atpI	210	TCA>TtA	S>L	83.72%	I-johSt	AJ271079.4	51384	C	T	43	0	-	0	36
				95.65%	II-suavG	EU262889.2	51311	C	T	69	0	-	0	66
				97.14%	III-graTus	KX014625.1	51338	C	T	70	0	-	0	68
matK	240	TCT>TtT	S>F	0%[3)]	I-johSt	AJ271079.4	2680	G	A	18	0	-	0	0
				61.53%	II-suavG	EU262889.2	2680	G	A	13	0	-	0	8
				71.42%	III-graTus	KX014625.1	2680	G	A	7	0	-	0	5
matK	396	CGG>tGG	R>W	40.00%	I-johSt	AJ271079.4	2213	G	A	35	12	0	-	2
				70.45%	II-suavG	EU262889.2	2213	G	A	44	31	0	-	0
				92.10%	III-graTus	KX014625.1	2213	G	A	38	35	0	-	0
ndhA	114	TCA>TtA	S>L	70.83%	I-johSt	AJ271079.4	127089	G	A	24	0	-	0	17
				80.00%	II-suavG	EU262889.2	126484	G	A	5	0	-	0	4
				n/a	III-graTus	KX014625.1	127953	G	A	0	0	-	0	0
ndhA	189	TCA>TtA	S>L	91.30%	I-johSt	AJ271079.4	125820	G	A	23	0	-	0	21
				100.00%	II-suavG	EU262889.2	125215	G	A	4	0	-	0	4
				100.00%	III-graTus	KX014625.1	126684	G	A	4	0	-	0	4
ndhA	358	TCC>TtC	S>F	42.85%	I-johSt	AJ271079.4	125313	G	A	28	0	-	0	12
				18.18%	II-suavG	EU262889.2	124708	G	A	11	0	-	0	2
				60.00%	III-graTus	KX014625.1	126177	G	A	5	0	-	0	3
ndhB	50	TCA>TtA	S>L	68.22%	I-johSt	AJ271079.4	103062	G	A	107	73	0	-	0
				59.61%	II-suavG	EU262889.2	102342	G	A	208	124	0	-	0
				89.47%	III-graTus	KX014625.1	103815	G	A	38	34	0	-	0
ndhB	156	CCA>CtA	P>L	76.19%	I-johSt	AJ271079.4	102744	G	A	21	16	0	-	0
				91.66%	II-suavG	EU262889.2	102024	G	A	36	33	0	-	0
				100.00%	III-graTus	KX014625.1	103497	G	A	5	5	0	-	0

Supplemental Table 2. (continued)

Gene	Codon Position	Codon	AA Exchange	Editing %	Plastome	Reference	Ref. Position	Ref. Base	Called Base	Depth	A Count	C Count	G Count	T Count
				33.33%	I-johSt	AJ271079.4	102625	G	A	12	4	0	-	0
ndhB	196	CAT>tAT	H>Y	70.00%	II-suavG	EU262889.2	101905	G	A	30	21	0	-	0
				57.14%	III-graTus	KX014625.1	103378	G	A	7	4	0	-	0
				25.00%	I-johSt	AJ271079.4	102600	G	A	16	4	0	-	0
ndhB	204	TCA>TtA	S>L	51.85%	II-suavG	EU262889.2	101880	G	A	27	14	0	-	0
				57.14%	III-graTus	KX014625.1	103353	G	A	7	4	0	-	0
				40.00%	I-johSt	AJ271079.4	102474	G	A	15	6	0	-	0
ndhB	246	CCA>CtA	P>L	76.82%	II-suavG	EU262889.2	101754	G	A	82	63	0	-	0
				80.00%	III-graTus	KX014625.1	103227	G	A	25	20	0	-	0
				30.00%	I-johSt	AJ271079.4	102465	G	A	20	6	0	-	0
ndhB	249	TCT>TtT	S>F	66.15%	II-suavG	EU262889.2	101745	G	A	65	43	0	-	0
				65.21%	III-graTus	KX014625.1	103218	G	A	23	13	0	-	0
				27.27%	I-johSt	AJ271079.4	101701	G	A	11	3	0	-	0
ndhB	277	TCA>TtA	S>L	37.83%	II-suavG	EU262889.2	100981	G	A	37	14	0	-	0
				25.00%	III-graTus	KX014625.1	102454	G	A	16	3	0	-	1
				27.27%	I-johSt	AJ271079.4	101695	G	A	11	3	0	-	0
ndhB	279	TCA>TtA	S>L	44.11%	II-suavG	EU262889.2	100975	G	A	34	15	0	-	0
				25.00%	III-graTus	KX014625.1	102448	G	A	16	4	0	-	0
				50.00%	I-johSt	AJ271079.4	101276	G	A	8	4	0	-	0
ndhB	419	CAT>tAT	H>Y	85.71%	II-suavG	EU262889.2	100556	G	A	28	24	0	-	0
				75.00%	III-graTus	KX014625.1	102029	G	A	4	3	0	-	0
				72.22%	I-johSt	AJ271079.4	101050	G	A	18	13	0	-	0
ndhB	494	CCA>CtA	P>L	74.00%	II-suavG	EU262889.2	100330	G	A	50	37	0	-	0
				91.66%	III-graTus	KX014625.1	101803	G	A	12	11	0	-	0
				9.09%	I-johSt	AJ271079.4	122817	G	A	22	2	0	-	0
ndhD	1	ACG>AtG	T>start	46.66%	II-suavG	EU262889.2	122015	G	A	15	7	0	-	0
				50.00%	III-graTus	KX014625.1	123480	G	A	2	1	0	-	0
				74.50%	I-johSt	AJ271079.4	122436	G	A	51	38	0	-	0
ndhD	128	TCA>TtA	S>L	62.50%	II-suavG	EU262889.2	121634	G	A	8	5	0	-	0
				40.00%	III-graTus	KX014625.1	123099	G	A	5	2	0	-	0

Supplemental Table 2. (continued)

Gene	Codon Position	Codon	AA Exchange	Editing %	Plastome	Reference	Ref. Position	Ref. Base	Called Base	Depth	A Count	C Count	G Count	T Count
ndhD	293	TCA>TtA	S>L	52.63%	I-johSt	AJ271079.4	121941	G	A	19	10	0	-	0
				80.00%	II-suavG	EU262889.2	121139	G	A	5	4	0	-	0
				83.33%	III-graTus	KX014625.1	122604	G	A	6	5	0	-	0
ndhD	296	CCT>CtT	P>L	24.00%	I-johSt	AJ271079.4	121932	G	A	25	6	0	-	0
				60.00%	II-suavG	EU262889.2	121130	G	A	5	3	0	-	0
				60.00%	III-graTus	KX014625.1	122595	G	A	5	3	0	-	0
ndhD	433	TCA>TtA	S>L	33.33%	I-johSt	AJ271079.4	121521	G	A	9	3	0	-	0
				94.73%	II-suavG	EU262889.2	120719	G	A	19	18	0	-	0
				64.28%	III-graTus	KX014625.1	122184	G	A	14	9	0	-	0
ndhK	22	TCA>TtA	S>L	80.95%	I-johSt	AJ271079.4	14298	C	T	42	0	-	0	34
				91.42%	II-suavG	EU262889.2	14290	C	T	70	0	-	0	64
				82.43%	III-graTus	KX014625.1	14321	C	T	74	0	-	0	61
pbf1	10	TCT>TtT	S>F	70.39%	I-johSt	AJ271079.4	79412	G	A	608	424	0	-	0
				21.87%	II-suavG	EU262889.2	78927	G	A	32	7	0	-	0
				13.33%	III-graTus	KX014625.1	80058	G	A	90	12	0	-	0
psaI	25	TCT>TtT	S>F	90.29%	I-johSt	AJ271079.4	64820	C	T	340	0	-	0	305
				90.81%	II-suavG	EU262889.2	64134	C	T	98	0	-	0	89
				94.04%	III-graTus	KX014625.1	65106	C	T	84	0	-	0	79
psaI	27	CAT>tAT	H>Y	76.50%	I-johSt	AJ271079.4	64825	C	T	332	0	-	0	254
				66.33%	II-suavG	EU262889.2	64139	C	T	101	0	-	0	67
				68.67%	III-graTus	KX014625.1	65111	C	T	83	0	-	0	57
psaI	31	AAC>AAt	N>N	34.50%	I-johSt	AJ271079.4	64839	C	T	313	0	-	1	107
				16.52%	II-suavG	EU262889.2	64153	C	T	115	0	-	0	19
				10.90%	III-graTus	KX014625.1	65125	C	T	110	0	-	0	12
psbF	26	TCT>TtT	S>F	75.00%	I-johSt	AJ271079.4	69928	G	A	536	370	0	-	0
				89.75%	II-suavG	EU262889.2	69297	G	A	166	133	0	-	0
				70.29%	III-graTus	KX014625.1	70229	G	A	606	385	1	-	0
psbZ	17	TCA>TtA	S>L	66.66%	I-johSt	AJ271079.4	29283	G	A	411	274	0	-	0
				81.27%	II-suavG	EU262889.2	29227	G	A	299	243	0	-	0
				73.68%	III-graTus	KX014625.1	29240	G	A	2379	1753	0	-	0

Supplemental Table 2. (continued)

Gene	Codon Position	Codon	AA Exchange	Editing %	Plastome	Reference	Ref. Position	Ref. Base	Called Base	Depth	A Count	C Count	G Count	T Count
rpl23	24	TCT>TtT	S>F	90.90%	I-johSt	AJ271079.4	91409	G	A	99	90	0	-	0
				97.34%	II-suavG	EU262889.2	90850	G	A	113	110	0	-	0
				95.23%	III-graTus	KX014625.1	91995	G	A	63	60	0	-	0
rpl23	30	TCA>TtA	S>L	82.82%	I-johSt	AJ271079.4	91391	G	A	99	82	0	-	0
				95.97%	II-suavG	EU262889.2	90832	G	A	149	142	0	-	0
				92.75%	III-graTus	KX014625.1	91977	G	A	69	64	0	-	0
rpoB	114	TCT>TtT	S>F	61.40%	I-johSt	AJ271079.4	39608	C	T	57	0	-	0	35
				72.22%	II-suavG	EU262889.2	39538	C	T	54	0	-	0	38
				75.00%	III-graTus	KX014625.1	39563	C	T	12	0	-	0	9
rpoB	185	TCA>TtA	S>L	80.76%	I-johSt	AJ271079.4	39821	C	T	26	0	-	0	21
				95.45%	II-suavG	EU262889.2	39751	C	T	66	0	-	0	63
				61.53%	III-graTus	KX014625.1	39776	C	T	13	0	-	0	8
rpoB	190	TCG>TtG	S>L	91.89%	I-johSt	AJ271079.4	39836	C	T	37	0	-	0	34
				98.33%	II-suavG	EU262889.2	39766	C	T	60	0	-	0	59
				61.53%	III-graTus	KX014625.1	39791	C	T	13	0	-	0	8
rpoC1	14	TCA>TtA	S>L	71.42%	I-johSt	AJ271079.4	42567	C	T	42	0	-	0	30
				77.27%	II-suavG	EU262889.2	42497	C	T	22	0	-	0	17
				63.63%	III-graTus	KX014625.1	42522	C	T	11	0	-	0	7
rpoC2	1375	CAA>tAA	Q>stop	54.54%	I-johSt	AJ271079.4	49581	C	T	11	0	-	0	6
				76.00%	II-suavG	EU262889.2	49511	C	T	25	0	-	0	19
				82.35%	III-graTus	KX014625.1	49536	C	T	17	0	-	0	14
rps12	74	TCA>TtA	S>L	92.15%	I-johSt	AJ271079.4	104754	G	A	2678	2467	0	-	0
				92.66%	II-suavG	EU262889.2	104034	G	A	4524	4187	0	-	0
				81.56%	III-graTus	KX014625.1	105507	G	A	868	708	0	-	0
rps12		intron		87.60%	I-johSt	AJ271079.4	104296	G	A	460	403	0	-	0
				92.39%	II-suavG	EU262889.2	103576	G	A	1709	1576	0	-	0
				95.17%	III-graTus	KX014625.1	105049	G	A	477	453	1	-	0
rps12		intron		83.82%	I-johSt	AJ271079.4	104443	G	A	303	251	0	-	0
				89.87%	II-suavG	EU262889.2	103723	G	A	5460	4879	0	-	1
				89.83%	III-graTus	KX014625.1	105196	G	A	1141	1017	0	-	0

Supplemental Table 2. (continued)

Gene	Codon Position	Codon	AA Exchange	Editing %	Plastome	Reference	Ref. Position	Ref. Base	Called Base	Depth	A Count	C Count	G Count	T Count
rps14	27	TCA>TtA	S>L	89.43%	I-johSt	AJ271079.4	27981	C	T	1013	0	-	0	905
				89.50%	II-suavG	EU262889.2	27927	C	T	1248	0	-	0	1117
				91.88%	III-graTus	KX014625.1	27943	C	T	2968	0	-	1	2726
rps14	50	TCA>TtA	S>L	86.31%	I-johSt	AJ271079.4	28050	C	T	694	0	-	0	599
				80.06%	II-suavG	EU262889.2	27996	C	T	1189	0	-	0	952
				86.91%	III-graTus	KX014625.1	28012	C	T	2086	0	-	0	1813
rps16		intron		89.73%	I-johSt	AJ271079.4	5549	G	A	3632	3257	0	-	2
				87.11%	II-suavG	EU262889.2	5554	G	A	1358	1182	1	-	0
				88.69%	III-graTus	KX014625.1	5525	G	A	115	102	0	-	0
rps2	45	ACA>AtA	T>I	91.54%	I-johSt	AJ271079.4	49962	C	T	71	0	-	0	65
				100.00%	II-suavG	EU262889.2	49889	C	T	25	0	-	0	25
				91.30%	III-graTus	KX014625.1	49916	C	T	23	0	-	0	21
ycf1	2348[4)]	ACC>ACt	T>T	40.55%	I-johSt	AJ271079.4	129743	G	A	2044	829	0	-	0
	2270[4)]			48.45%	II-suavG	EU262889.2	129138	G	A	1232	595	2	-	0
	2325[4)]			68.09%	III-graTus	KX014625.1	130577	G	A	652	444	0	-	0

[1)] Keuthe [24] identified an additional site in *ndhG* (S17L) that is conserved between *Oenothera*, tobacco and *Arabidopsis*. In the present study, editing of this site was confirmed in I-johSt, but found to be below the chosen threshold of 30%. Furthermore, we could not detect edited reads in our II-sauvG and III-graTusa data sets, whereas Keuthe reported 69% and 48% editing of this site for I-hookdV and III-lamS, respectively.

[2)] For genes nested within the inverted repeat (IR), and therefore present twice in the plastome, only the IR_B positions are provided.

[3)] Site confirmed with 73% editing efficiency by Keuthe [24] in I-hookdV.

[4)] Due to large indels within the *ycf1* gene, the codon position of this site varies among the *Oenothera* species.

Supplemental Table 3. Origin and collector information for the *Oenothera* strains used in this work.

Species	Strain	Locality	Collection date	Collector	Reference
O. elata ssp. *elata*	elata Cholula	Mexico, Puebla, 6 miles north-west of Cholula de Rivadavia	before 1949	P. A. Munz	[25,26]
O. elata ssp. *elata*	elata Guatemala	Guatemala, Guatemala, Guatemala City	1945	P. Weatherwax	[27]
O. elata ssp. *elata*	elata Puebla	Mexico, Puebla, garden at Puebla	before 1949	P. A. Munz	[25,26]
O. elata ssp. *elata*	elata Toluca	Mexico, Mexico, garden at Toluca de Lerdo	1937	P. A. Munz	[25,27]
O. elata ssp. *hookeri*	franciscana de Vries[1)]	USA, CA, Monterey Co., Carmel Beach	1905	C. P. Smith	[28,29]
O. elata ssp. *hookeri*	hookeri de Vries	USA, CA, Alameda Co., near Berkeley	1904	H. de Vries	[30]
O. elata ssp. *hookeri*	johansen Standard	USA, CA, Sutter Co., roadside between Nicholas and Yuba City	1927	C. B. Wolf	[31]
O. villosa ssp. *strigosa*	strigosa de Vries	USA, WY, Park Co., Yellowstone National Park near Mammoth Hot Springs	1904	H. de Vries	[30]
O. villosa ssp. *villosa*	bauri Standard	Poland, Kujawsko-Pomorskie, near Toruń	before 1942	R. Hölscher	[32]
O. villosa ssp. *villosa*	cockerelli de Vries	USA, CO, Boulder Co., near Boulder	1905	T. D. A. Cockerell	[30]
O. villosa ssp. *villosa*	mollis Standard	Germany, Brandenburg, in sandy soil near Jüterbog	1934	O. Renner	[33]
O. villosa ssp. *villosa*	strigosa Iowa 2	USA, IA, Dickson Co., in a gravel pit one mile south-west of Manhattan Beach	1930	J. B. Eisen	[34]
O. villosa ssp. *villosa*	strigosa Iowa 6	USA, IA, Dickson Co., on the slope of a gravel pit one mile south-west of Manhattan Beach	1930	J. B. Eisen	[35]
O. villosa ssp. *villosa*	villosa Madeleine	Canada, QC, Gaspésie-Îles-de-la-Madeleine Magdalen Islands	before 1967	Anonymous	[20]
O. wolfii	wolfii Crescent City 1	USA, CA, Del Norte Co., Crescent City near marina	1977	P. C. Hoch	[20,21]
O. wolfii	wolfii Crescent City 3	USA, CA, Del Norte Co., Crescent City near marina	1977	P. C. Hoch	[20,21]
O. wolfii	wolfii Lufftenholtz	USA, CA, Humboldt Co., Luffenholtz, Beach County Park south of Trinidad	1975	J. D. Ackerman and A. M. Montalvo	[20,21]
O. wolfii	wolfii Petrolia	USA, CA, Humboldt Co., Petrolia	1977	P. C. Hoch	[20]

Supplemental Table 3. (continued)

Species	Strain	Locality	Collection date	Collector	Reference
O. biennis x glazioviana	conferta Standard	France, Calvados, near Cabourg north-east of Caen, directly on a costal sand dune	before/in 1942	F. Hilpert	[36]
O. biennis x glazioviana	coronifera Standard	Germany, Brandenburg, railway embankment at Zinna Abbey near Jüterbog	1936	O. Renner	[37]
O. biennis x villosa	hoelscheri Standard	Poland, Kuyavian-Pomeranian, near Vistula River at Włocławek	before 1942	R. Hölscher	[38]
O. biennis	biennis Muenchen	Germany, Bavaria, Munich, Nymphenburg Garden	1914	O. Renner	[39]
O. biennis	biennis Shuswap Lake	Canada, BC, Shuswap Lake	1977	G. B. Straley	[20]
O. biennis	nuda Standard	France, Isère, narrow-gauge railway embankment at both sides of Saint-Laurent-du-Pont	1947	A. Gagnieu	[38]
O. biennis	purpurata Standard[2)]	chromosome translocation mutant of material reassembling biennis *cruciata* Klebahn[3)]	isolated in 1914	H. Klebahn	[40,41]
O. biennis	rubricaulis Thorn	Poland, Kujawsko-Pomorskie, Vistula River near Toruń	before 1941	R. Hölscher	[42]
O. biennis	suaveolens Standard	France, Seine-et-Marne, forest of Fontainebleau	1912	L. Blaringhem	[43]
O. biennis	suaveolens Grado	Italy, Friuli-Venezia Giulia, dune near Grado at the Adriatic sea	before 1950	H. Zeidler	[44,45]
O. biennis	suaveolens *xanthodermis* Fuenfkirchen	Hungary, Baranya, near Pécs	before 1949	E. Preuss	[44,45]
O. biennis	suaveolens *sulfurea* Friedrichshagen	Germany, Berlin, Treptow-Köpenick, at railway station Friedrichshagen-Hirschgarten	1937	O. Renner	[44,45]
O. biennis	chicaginensis Colmar	France, Haut-Rhin, fallow on the road between Niederhergheim and mill at Dessenheim	1943	E. Issler	[38]
O. biennis	chicaginensis de Vries	USA, IL, Cook Co., Chicago, near Jackson Park	1904	H. de Vries	[30]
O. biennis	biennis-1 Citronelle	USA, AL, Mobile Co., Citronelle	1935	P. A. Munz	[46]
O. biennis	biennis-1 Hot Springs	USA, AR, Garland Co., Hot Springs	before 1958	Anonymous	[47]

Supplemental Table 3. (continued)

Species	Strain	Locality	Collection date	Collector	Reference
O. biennis	biennis-1 Paducah	USA, KY, McCracken Co., eleven miles west of Paducah	1935	P. A. Munz	[34]
O. biennis	biennis-1 Walkerton	USA, IN, St. Joseph Co., Walkerton	before 1958	Anonymous	[47]
O. glazioviana	*r*/*r*-lamarckiana Sweden	Sweden, Skåne Län, garden in Almaröd	1906	N. Heribert-Nilsson	[48]
O. grandiflora	grandiflora Tuscaloosa	USA, AL, Mobile Delta	1944	J. S. Lloyd	[49]
O. oakesiana	ammophila Standard	Germany, Schleswig-Holstein, Helgoland	1922	E. Hoeppener	[50]
O. oakesiana	ammophila Sylt	Germany, Schleswig-Holstein, southern tip of the island of Sylt	before 1963	Anonymous	[8]
O. oakesiana	germanica Standard	Germany, Berlin, Berlin-Rahnsdof	1918	E. Baur	[50,51]
O. oakesiana	parviflora-1 Iron Mountain[4)]	USA, MI, Dickinson Co., five miles south-east of Iron Mountain	1938	P. A. Munz	[52]
O. oakesiana	*r*/*r*-syrticola Ulm	Germany, Baden-Württemberg, Danube River near Ulm	1917	O. Renner	[33]
O. parviflora	atrovirens Standard[5)]	USA, NY, Erie Co., Sandy Hill near Lake George	1902/1903	D. T. MacDouglas	[30,53]
O. parviflora	silesiaca Standard	Poland, Dolnośląskie, bank of Bóbr River near Nowogrodziec	1937	O. Renner	[42]
O. parviflora	rubricuspis Standard	Germany, Hessen, railway embankment between Neu-Isenburg and Luisa near Frankfurt on the Main	1942/1943	O. Burck, F. Laibach and E. Fischer	[45]

[1)] The strain franciscana de Vries is a derivative of Davis's franciscana B [54], as summarized in Davis (1916) [55].

[2)] Originally described by Klebahn as *Oenothera biennis rubicalyx* [40].

[3)] Derivative of parent plant No. 347; similar to biennis *cruciata* Klebahn collected from "Population 4" near Bad Bevensen (Germany, Niedersachsen) [40].

[4)] This line was originally described as parviflora-1 (= BC-IV = *O. parviflora*) by Cleland [56], but identified as AC-IV (= parviflora-2 = *O. oakesiana*) by Wasmund [20].

[5)] According to Renner [53] this line was originally "received from Amsterdam" by N. v. Gescher in 1907. The material is quite likely identical to that collected by D. T. MacDouglas in 1902/1903 as described in de Vries (1913) [30]. Also see Bartlett (1914) [57].

Supplemental Table 4. Chloroplast substitution lines, F1 hybrids, and corresponding wild types used in this work.

Line and genotype	Type	Plastome	Nuclear genome[1)]	Chloroplast donor strain[2)]	Nucleus donor strain(s)[2)]	Phenotype	Produced by	Reference
AA-I	wild type	I-johSt	^{h}*johansen Standard*· ^{h}*johansen Standard*	johansen Standard	johansen Standard	green	wild type	[9,14,16,31]
AA-II	substitution line	II-suavG	^{h}*johansen Standard*· ^{h}*johansen Standard*	suaveolens Grado	johansen Standard	green	W. Stubbe	[11,58], this work
AB-I	substitution line	I-johSt	^{G}*albicans*·^{G}*flavens*	johansen Standard	suaveolens Grado	*lutescent*	S. Greiner	this work
AB-I	F1 hybrid	I-johSt	^{h}*johansen Standard*· ^{h}*grandiflora Tuscaloosa*	johansen Standard	johansen Standard & grandiflora Tuscaloosa	*lutescent*	S. Greiner	[19,59]
AB-II	wild type	II-suavG	^{G}*albicans*·^{G}*flavens*	suaveolens Grado	suaveolens Grado	green	wild type	[12,13,44]
AB-II	F1 hybrid between substitution line and wild type	II-suavG	^{h}*johansen Standard*· ^{h}*grandiflora Tuscaloosa*	suaveolens Grado	johansen Standard & grandiflora Tuscaloosa	green	S. Greiner	this work

1) See Methods for details.

2) For details on the donor stains, see Supplemental Table 3.

Supplemental Table 5. Oligonucleotides used for the generation of probes for RNA gel blot and run-on transcription analyses.

Name	Gene	Sequence (5' to 3')
Probes for RNA gel blot analyses		
AZpsbBNorth_for	*psbB*	TTTTCTGATGAACGCACAGG
AZpsbBNorth_rev	*psbB*	TAATACGACTCACTATAGGGTCCAGCAACAACAAAAGCTG
AZpsbTNorth_for	*psbT*	GGAAGCATTGGTTTATACATTCC
AZpsbTNorth_rev	*psbT*	TAATACGACTCACTATAGGGTGAATTTTAGGCGGTTCTCG
AZpsbHNorth_for	*psbH*	GGCTACACAAACTGCTGAGG
AZpsbHNorth_rev	*psbH*	TAATACGACTCACTATAGGGTCCGTCCAATAAAACGGAAG
AZpetBNorth_for	*petB*	GGTCGGCAAGTATGATGGTC
AZpetBNorth_rev	*petB*	TAATACGACTCACTATAGGGCCAGAAATCCCTTGCTTACG
AZpetDNorth_for	*petD*	AACCATCAATGCTTGGTGAAC
AZpetDNorth_rev	*petD*	TAATACGACTCACTATAGGGAAGACCTAAGGTTAGGGATTTATCG
1psbN_N_F	*pbf1*	GGAAACAGCAACCCTAGTCG
1psbN_N_R	*pbf1*	TAATACGACTCACTATAGGGGTGTTCCTCGAACGGATCTC
AZpsbMNorth_for	*psbM*	TGGGAAGTAAATATTCTCGCATTTATTG
AZpsbMNorth_rev	*psbM*	TAATACGACTCACTATAGGGTCACTTTGACTGACAGTTTTTACG
1petN_N_F	*petN*	TGGATATAGTCAGTCTTGCTTGG
1petN_N_R	*petN*	TAATACGACTCACTATAGGGCCACTCCTTCCCCATACTACC
1clpP_N_F	*clpP*	CTTTTTAGGCGACGCAATTC
1clpP_N_R	*clpP*	TAATACGACTCACTATAGGGTAGGCGTTTGGACGTTTCTC
AZ5rps12_for	*rps12*	ACACAAGACAGCCAATCAG
AZ5rps12_rev	*rps12*	TAATACGACTCACTATAGGGCACCCTAGTACATGTTCCTC
AZrpl20_for	*rps20*	GCTTGGTTTTCGTCTCATCG
AZrpl20_rev	*rpl20*	TAATACGACTCACTATAGGGGCGGATTCTTGCCCAATCTAC
Probes for run-on DNA probes		
psbBRO_F	*psbB*	CTAATTCATGGGGTGGTTGG
psbBRO_R	*psbB*	AAGAGCAGAGCAAACGAAGC
petBRO_F	*petB*	TCTCGAGATTCAGGCGATTG
petBRO_R	*petB*	CCAGAAATCCCTTGATTACG
16Sfor_Sonde_MK	*16S rRNA*	GAAAGAGAGGTGTGCCTTCG
16Srev_Sonde_MK	*16S rRNA*	TAATACGACTCACTATAGGGTACTTCATGCAGGCGAGTTG
23for_Sonde_MK	*23S rRNA*	TGCCATACTCCCAGGAAAAG
23rev_Sonde_MK	*23S rRNA*	TAATACGACTCACTATAGGGTTACCCGACAAGGAATTTCG

SUPPLEMENTAL REFERENCES

1 **Schöttler MA, Tóth SZ.** 2014. Photosynthetic complex stoichiometry dynamics in higher plants: environmental acclimation and photosynthetic flux control. *Frontiers in Plant Science* **5**: 188.

2 **Krause GH, Weis E.** 1991. Chlorophyll fluorescence and photosynthesis: the Basics. *Annual Review of Plant Physiology and Plant Molecular Biology* **42**: 313-49.

3 **Albus CA, Ruf S, Schöttler MA, Lein W, et al.** 2010. Y3IP1, a nucleus-encoded thylakoid protein, cooperates with the plastid-encoded Ycf3 protein in photosystem I assembly of tobacco and *Arabidopsis*. *The Plant Cell* **22**: 2838-55.

4 **Krech K, Ruf S, Masduki FF, Thiele W, et al.** 2012. The plastid genome-encoded Ycf4 protein functions as a nonessential assembly factor for photosystem I in higher plants. *Plant Physiology* **159**: 579-91.

5 **Rott M, Martins NF, Thiele W, Lein W, et al.** 2011. ATP synthase repression in tobacco restricts photosynthetic electron transport, CO_2 assimilation, and plant growth by overacidification of the thylakoid lumen. *The Plant Cell* **23**: 304-21.

6 **Cruz JA, Sacksteder CA, Kanazawa A, Kramer DM.** 2001. Contribution of electric field ($\Delta\psi$) to steady-state transthylakoid proton motive force (*pmf*) *in vitro* and *in vivo*. Control of *pmf* parsing into $\Delta\psi$ and ΔpH by Ionic strength. *Biochemistry* **40**: 1226-37.

7 **Westhoff P, Herrmann RG.** 1988. Complex RNA maturation in chloroplasts. The *psbB* operon from spinach. *European Journal of Biochemistry* **171**: 551-64.

8 **Stubbe W.** 1963. Die Rolle des Plastoms in der Evolution der Oenotheren. *Berichte der Deutschen Botanischen Gesellschaft* **76**: 154-67.

9 **Drillisch M.** 1975. Vergleichende Untersuchungen an "A-Genotypen" von *Oenothera* [PhD Thesis]. Heinrich Heine University Düsseldorf.

10 **Dietrich W, Wagner WL, Raven PH.** 1997. Systematics of *Oenothera* section *Oenothera* subsection *Oenothera* (Onagraceae). *Systematic Botany Monographs* **50**: 1-234.

11 **Massouh A, Schubert J, Yaneva-Roder L, Ulbricht-Jones ES, et al.** 2016. Spontaneous chloroplast mutants mostly occur by replication slippage and show a biased pattern in the plastome of *Oenothera*. *The Plant Cell* **28**: 911-29.

12 **Stubbe W.** 1960. Untersuchungen zur genetischen Analyse des Plastoms von *Oenothera*. *Zeitschrift für Botanik* **48**: 191-218.

13 **Stubbe W.** 1959. Genetische Analyse des Zusammenwirkens von Genom und Plastom bei *Oenothera*. *Zeitschrift für Vererbungslehre* **90**: 288-98.

14 **Cleland RE.** 1962. Plastid behaviour of the North American Euoenotheras. *Planta* **57**: 699-712.

15 **Sobanski J, Giavalisco P, Fischer A, Kreiner JM, et al.** 2019. Chloroplast competition is controlled by lipid biosynthesis in evening primroses. *Proceedings of the National Academy of Sciences* **116**: 5665-74.

16 **Kozul D.** 2019. Systematic identification of loci determining chloroplast and nuclear genome incompatibilities in the evening primrose (*Oenothera*) [PhD Thesis]. University of Potsdam.

17 **Hupfer H, Swiatek M, Hornung S, Herrmann RG, et al.** 2000. Complete nucleotide sequence of the *Oenothera elata* plastid chromosome, representing plastome I of the five distinguishable *Euoenothera* plastomes. *Molecular and General Genetics* **263**: 581-5.

18 **Greiner S, Wang X, Rauwolf U, Silber MV, et al.** 2008. The complete nucleotide sequences of the five genetically distinct plastid genomes of *Oenothera*, subsection *Oenothera*: I. Sequence evaluation and plastome evolution. *Nucleic Acids Research* **36**: 2366-78.

19 **Greiner S, Wang X, Herrmann RG, Rauwolf U, et al.** 2008. The complete nucleotide sequences of the 5 genetically distinct plastid genomes of *Oenothera*, subsection *Oenothera*: II. A microevolutionary view using bioinformatics and formal genetic data. *Molecular Biology and Evolution* **25**: 2019-30.

20 **Wasmund O.** 1980. Cytogenetische Untersuchung zur Systematik einiger Sippen der Subsektion *Euoenothera* der Gattung *Oenothera* (*Onagraceae*) [State Examination Thesis]. Heinrich Heine University Düsseldorf.

21 **Wasmund O, Stubbe W.** 1986. Cytogenetic investigations on *Oenothera wolfii* (Onagraceae). *Plant Systematics and Evolution* **154**: 79-88.

22 **Schumacher E, Steiner EE, Stubbe W.** 1992. The complex-heterozygotes of *Oenothera grandiflora* L'Her. *Botanica Acta* **105**: 375-81.

23 **Hupfer H.** 2002. Vergleichende Sequenzanalyse der fünf Grundplastome der Sektion *Oenothera* (Gattung *Oenothera*) - Analyse des Cytochrom-Komplexes [PhD Thesis]. Ludwig Maximilian University of Munich.

24 **Keuthe M.** 2013. Identifizierung und Charakterisierung von Suppressormutanten einer Plastom-Genom-Inkompatibilität in *Oenothera* [PhD Thesis]. University of Potsdam.

25 **Munz PA.** 1949. The *Oenothera hookeri* group. *El Aliso* **2**: 1-47.

26 **Steiner EE.** 1955. A cytogenetic study of certain races of *Oenothera elata*. *Bulletin of the Torrey Botanical Club* **82**: 292-7.

27 **Steiner EE.** 1951. Phylogenetic relationships of certain races of *Euoenothera* from Mexico and Guatemala. *Evolution* **5**: 265-72.

28 **Cleland RE.** 1922. The reduction divisions in the pollen mother cells of *Oenothera franciscana*. *American Journal of Botany* **9**: 391-413.

29 **Bartlett HH.** 1914. Systematic studies on *Oenothera*: IV. *Oe. franciscana* and *Oe. venusta*, spp. novv. *Rhodora* **16**: 33-7.

30 **de Vries H.** 1913. Gruppenweise Artbildung - Unter spezieller Berücksichtigung der Gattung *Oenothera* Berlin: Gebrüder Borntraeger.

31 **Cleland RE.** 1935. Cyto-taxonomic studies on certain Oenotheras from California. *Proceedings of the American Philosophical Society* **75**: 339-429.

32 **Baerecke M.** 1944. Zur Genetik und Cytologie von *Oenothera ammophila* Focke, *Bauri* Boedijn, *Beckeri* Renner, *parviflora* L., *rubricaulis* Klebahn, *silesiaca* Renner. *Flora* **138**: 57-92.

33 **Renner O.** 1937. Wilde Oenotheren in Norddeutschland. *Flora* **31**: 182-226.

34 **Cleland RE, Hammond BL.** 1950. Anaylsis of segmental arragenments in certain race of *Oenothera*. In Cleland RE (ed) *Studies in Oenothera cytogenetics and phylogeny*. *Indiana University Publications. Science Series* **16**: 10-72.

35 **Cleland RE.** 1937. Species relationships in *Onagra*. *Proceedings of the American Philosophical Society* **77**: 477-542.

36 **Renner O, Hirmer U.** 1956. Zur Kenntnis von *Oenothera*: I. Über *Oe. conferta* n. sp. II. Über künstliche Polyploidie. *Biologisches Zentralblatt* **75**: 513-31.

37 **Rossmann G.** 1963. Analyse der *Oenothera coronifera* Renner. *Flora* **153**: 451-68.

38 **Renner O.** 1956. Europäische Wildarten von *Oenothera*: III. *Planta* **47**: 219-54.

39 **Renner O.** 1917. Versuche über die gametische Konstitution der Önotheren. *Zeitschrift für induktive Abstammungs- und Vererbungslehre* **18**: 121-294.

40 **Klebahn H.** 1914. Formen, Mutationen, und Kreuzungen bei einigen Oenotheren aus der Lüneburger Heide. *Jahrbuch der Hamburger Wissenschaftlichen Anstalten* **31**: 1-64.
41 **Klebahn H.** 1925. Weitere Beobachtungen über Oenotheren aus Nordwestdeutschland. *Zeitschrift für induktive Abstammungs- und Vererbungslehre* **39**: 8-30.
42 **Renner O.** 1943. Kurze Mitteilung über *Oenothera*: V. Zur Kenntnis von *O. silesiaca* n. sp., *parviflora* L., *ammophila* Focke, *rubricaulis* Kleb. *Flora* **36**: 325-35.
43 **de Vries H.** 1918. Mutations of *Oenothera suaveolens* Desf. *Genetics* **3**: 1-26.
44 **Stubbe W.** 1953. Genetische und zytologische Untersuchungen an verschiedenen Sippen von *Oenothera suaveolens*. *Zeitschrift für induktive Abstammungs- und Vererbungslehre* **85**: 180-209.
45 **Renner O.** 1950. Europäische Wildarten von *Oenothera*: II. *Berichte der Deutschen Botanischen Gesellschaft* **63**: 129-38.
46 **Steiner E.** 1952. Phylogenetic studies in *Euoenothera*. *Evolution* **6**: 69-80.
47 **Cleland RE.** 1958. The evolution of the North American Oenotheras of the "*biennis*" group. *Planta* **51**: 378-98.
48 **Heribert-Nilsson N.** 1912. Die Variabilität der *Oenothera Lamarckiana* und das Problem der Mutation. *Zeitschrift für induktive Abstammungs- und Vererbungslehre* **8**: 89-231.
49 **Steiner EE, Stubbe W.** 1984. A contribution to the population biology of *Oenothera grandiflora* L'Her. *American Journal of Botany* **71**: 1293-301.
50 **Hoeppener E, Renner O.** 1929. Genetische und zytologische Oenotherenstudien: I. Zur Kenntnis der *Oenothera ammophila* Focke. *Zeitschrift für induktive Abstammungs- und Vererbungslehre* **49**: 1-25.
51 **Boedijn K.** 1924. Die systematische Gruppierung der Arten von Oenothera. *Zeitschrift für induktive Abstammungs- und Vererbungslehre* **32**: 354-62.
52 **Geckler H.** 1950. The nature and ralationships of taxonomic events in the North American *Euoenotheras*. In Cleland RE (ed) *Studies in Oenothera cytogenetics and phylogeny*. *Indiana University Publications. Science Series*. **16**: 160-217.
53 **Renner O.** 1938. Über *Oenothera atrovirens* Sh. et Bartl. und über somatische Konversion im Erbgang des *cruciata*-Merkmals der Oenotheren. *Zeitschrift für induktive Abstammungs- und Vererbungslehre* **74**: 91-124.
54 **Davis BM.** 1916. Hybrids of *Oenothera biennis* and *Oenothera franciscana* in the first and second generations. *Genetics* **1**: 197-251.
55 **Renner O.** 1941. Über die Entstehung homozygotischer Formen aus kompelx-heterozygotischen Oenotheren. *Flora* **135**: 201-38.
56 **Cleland RE.** 1972. *Oenothera* - Cytogenetics and Evolution London, New York: Academic Press Inc.
57 **Bartlett HH.** 1914. An account of the cruciate-flowered Oenotheras of the subgenus *Onagra*. *American Journal of Botany* **1**: 226-43.
58 **Stubbe W.** 1989. *Oenothera* - An ideal system for studying the interaction of genome and plastome. *Plant Molecular Biology Reporter* **7**: 245-57.
59 **Rauwolf U, Golczyk H, Meurer J, Herrmann RG, et al.** 2008. Molecular marker systems for *Oenothera* genetics. *Genetics* **180**: 1289-306.
